# Identification of *FCER1G* related to Activated Memory CD4^+^ T Cells Infiltration by Gene Co-expression Network and Construction of a Risk Prediction Module in Diffuse Large B-Cell Lymphoma

**DOI:** 10.3389/fgene.2022.849422

**Published:** 2022-05-30

**Authors:** Xiaoyu Xiang, Li-Min Gao, Yuehua Zhang, Yuan Tang, Sha Zhao, Weiping Liu, Yunxia Ye, Wenyan Zhang

**Affiliations:** Department of Pathology, West China Hospital of Sichuan University, Chengdu, China

**Keywords:** diffuse large B-cell lymphoma, weighted gene co-expression network analysis, memory activated CD4^+^ T cells, prognosis biomarkers, FCER1G

## Abstract

Diffuse large B cell lymphoma (DLBCL) is a group of biologically heterogeneous tumors with different prognoses. The tumor microenvironment plays a vital role in the tumorigenesis and development of DLBCL, and activated memory CD4^+^ T cells are an essential component of immunological cells in the lymphoma microenvironment. So far, there are few reports about activated memory CD4+T cells infiltration and related genes in the DLBCL tumor microenvironment. This study obtained the mRNA expression profile information of the testing GSE87371 dataset and another six validation datasets (GSE53786, GSE181063, GSE10846, GSE32918, GSE32018, GSE9327, GSE3892, TCGA-DLBC) from the GEO and TCGA databases. Weighted Gene Co-expression Network Analysis (WGCNA) screened gene module associated with activated memory CD4^+^ T cells infiltration. CIBERSORT and TIMER (immune cells infiltrating estimation analysis tools) were used to identify the relationship between activated memory CD4^+^ T cells and genes associated with immune infiltrating cells in the tumor microenvironment. The least absolute shrinkage and selection operator (LASSO) built the risk prediction model and verified it using nomogram and Kaplan-Meier analysis. Further functional characterization includes Gene Ontology, KEGG pathway analysis and Gene Set Enrichment Analysis (GSEA) to investigate the role and underlying mechanisms of these genes. These results suggest that the expression of *FCER1G* can reflect the invasion of activated memory CD4^+^ T cells in DLBCL, which provides a new idea for studying the tumor microenvironment and may become a potential predictive biomarker for the assessment of DLBCL.

## Introduction

Diffuse large B-cell lymphoma (DLBCL), the most common entity of non-Hodgkin’s lymphoma (NHL) (Chambwe et al., 2014), is a group of heterogeneous diseases with diverse prognoses (Risueño et al., 2020), accounting for 30–40% of newly diagnosed lymphoma (Trinh et al., 2013). Unfortunately, despite the addition of the anti-CD20 monoclonal antibody rituximab (R) to standard chemotherapy [e.g., “cyclophosphamide, doxorubicin, vincristine, and prednisone (R-CHOP)”], 30–40% of patients still relapse (10% of patients with refractory) (Raut and Chakrabarti, 2014). Recently, immune checkpoint inhibitors have provided an alternative way in the first-line treatment of tumors (Atanackovic and Luetkens, 2018; Wang et al., 2020). However, there are few specific molecular markers for DLBCL immunotherapy (Lossos and Morgensztern, 2006). The exploration of immune-related molecular markers is an essential research hotspot in DLBCL.

Immune invasion of DLBCL has been reported in many studies (Xu-Monette et al., 2019; Autio et al., 2021), and changes in tumor microenvironment can affect the response to immunotherapy (Rosato et al., 2019). Previous studies have focused on cytolysis CD8^+^ T cells as tumor-infiltrating lymphocytes (Felgar et al., 1998). However, some studies have recently demonstrated that CD4^+^ T cells are critical mediators of peripheral tolerance and immunosuppression and may play a central role in anti-tumor immunity. In addition, activation of CD4^+^ T cells in DLBCL has been reported to indicate a better prognosis (Keane et al., 2013; Kusano et al., 2017), but the mechanism remains unclear. Therefore, identifying biomarkers related to CD4^+^ T cell infiltration is conducive to monitoring DLBCL immunotherapy response and exploring the mechanism of immune infiltration.

With the development of the high-throughput sequencing technique, numerous tools for detecting disease biomarkers have emerged. Weighted gene co-expression network analysis (WGCNA) is applied to search for gene modules of co-expression genes and explore the relationship between gene networks and focused phenotypes. Cell type identification by estimating the relative subset of RNA transcripts (CIBERSORT) is another bioinformatics tool for analyzing gene expression data, which is a method for characterizing the cell composition of complex tissue by deconvolution method (Newman et al., 2015). Tumor immune estimation resource (TIMER) is an updated webserver with unique features that enable analysis and visualization of tumor molecular and clinical features. Based on the above computer tools and algorithms, we can comprehend the approximate cell types and amounts of immune cell infiltration.

In this study, WGCNA was performed using DLBCL gene expression data to search for gene modules highly associated with activated memory CD4^+^ T cells infiltration in order to explore the impact of the tumor microenvironment and identify potential biomarkers of DLBCL. The compositions of immune cells were calculated by the CIBERSORT algorithm. We identified essential modules and hub genes relevant to activated memory CD4^+^ T lymphocytes infiltration level and immune and clinical features of these hub genes. LASSO Cox regression module was used to identify and verify the predictive biomarkers. As we known, this study is the first utilization of WGCNA to identify the biomarkers related to activated memory CD4^+^ T lymphocytes of DLBCL.

## Materials and Methods

### Collecting RNA Expression Data From Gene Expression Omnibus Databases

Datasets were downloaded in a normalized expression matrix file format and analyzed directly from Gene Expression Omnibus (GEO; http://www.ncbi.nlm.nih.gov/geo/) and The Cancer Genome Atlas (TCGA; https://portal.gdc.cancer.gov/), which is an international public repository containing high-throughput microarray and next-generation sequencing functional genomic datasets (Barrett et al., 2013). All samples from DLBCL GEO cohorts, cases in the four datasets were divided into testing group GSE87371 (Dubois et al., 2017) and validation group, including GSE181063 (Painter et al., 2019; Lacy et al., 2020), GSE53786 (Scott et al., 2014), GSE10846 (Lenz et al., 2008), GSE32918 (Barrans et al., 2012), GSE32018 (Gómez-Abad et al., 2011), GSE9327 (Ruiz-Vela et al., 2008), GSE3892 (Muris et al., 2007) and TCGA-DLBC. The platform used by GSE87371 is [HG-U133_Plus_2] Affymetrix Human Genome U133 Plus 2.0 Array, which includes 223 DLBCL tumor tissue samples. The platform used by GSE181063 is Illumina HumanHT-12 WG-DASL V4.0 R2 expression beadchip, which includes 1303 DLBCL tumor tissue samples. The platform used by GSE53786 is [HG-U133_Plus_2] Affymetrix Human Genome U133 Plus 2.0 Array, which includes 119 DLBCL tumor tissue samples. The platform used by GSE10846 is [HG-U133 Plus 2] Affymetrix Human Genome U133 Plus 2.0 Array, which includes 412 DLBCL tumor tissue samples. The platform used by GSE32918 is Illumina HumanRef-8 WG-DASL v3.0, which includes 243 DLBCL tumor tissue samples. The platform used by GSE32018 is Agilent-014850 Whole Human Genome Microarray 4x44K G4112F (Probe Name version), which includes 22 DLBCL tumor tissue samples and 13 normal tissue samples. The platform used by GSE9327 is CNIO Human Oncochip 1.0, 1.2, and 2.0, which includes 36 DLBCL tumor tissue samples and 8 normal tissue samples. The platform used by GSE3892 is VUMC MACF human 19K oligo v33, which includes 52 DLBCL tumor tissue samples and 2 normal tissue samples. TCGA-DLBC contains 48 DLBCL tumor tissue samples. We used the R package “limma” (Ritchie et al., 2015) (https://cran.r-project.org/src/contrib/Archive/limma/) to normalize the RNA-sequencing data. Since the slight variation of gene expression data often makes noise, we used the Coefficient of Variation (CV) values to select the most variant genes and then construct the network.

### Identifying of Immune-Infiltrating Immune Cells (TIICs) by CIBERSORT

CIBERSORT (http://cibersort.stanford.edu) is an analytical algorithm that analyzes RNA expression data to assess the abundance of different cell subtypes for each sample (Newman et al., 2015). The fraction of TIICs was calculated using the R package “CIBERSORT” in the research. The proportion of seven subtypes of T cells in each sample was selected to analyze.

### Constructing Co-expression Network of Activated Memory CD4^+^ T Cells Infiltration in DLBCL

The “WGCNA” R package (https://cran.r-project.org/web/packages/WGCNA/index.html) was applied to reveal correlations between genes (Langfelder and Horvath, 2008). We calculated the average connectivity and Pearson correlation value to cluster GSE87371 samples. Next, we screened the genes with the first 75% of the median absolute deviation (MAD) and set the cut-off >0.01. The selected genes were used to construct the weighted co-expression network analysis. To figure out the gene module associated with activated memory CD4^+^ T cells infiltration, we built a scale-free network and picked *ß* = 5 as the soft-thresholding power. The hierarchical clustering dendrogram summarized the gene modules with different colors using dynamic hybrid cutting. Eventually, the heat map and topological overlap matrix (TOM) plot visualized the module structures.

### Selecting the Key Gene Module by the Relationship of Traits and Modules

Module eigengenes were used to analyze the components of each module. We calculated the correlation between the module eigengenes and T cells infiltration number to determine the significance of the module by Pearson’s test. An individual module was considered to be significantly associated with T cells infiltration when *p*-value <0.05. A module of T lymphocyte subtypes that demonstrated a high correlation coefficient was defined as the hub module.

### Analyzing the Functional Enrichment Pathways and Processes

Gene functions were distinguished into three aspects by Gene Ontology (GO) analysis, including Molecular Function (MF), Cellular Component (CC), and Biological Process (BP) (Harris et al., 2004). Molecular pathway maps were collected by the Kyoto Encyclopedia of Genes and Genomes (KEGG), which represented molecular interactions and reaction networks. They were divided into seven categories: metabolism, genetic and environmental information processing, cellular process, body system, human disease, and drug development (Kanehisa and Goto 2000). The results were displayed by the “clusterProfiler” (Yu et al., 2012) (https://bioconductor.org/packages/release/bioc/html/clusterProfiler.html) and “GOplot” R packages (Walter et al., 2015) (https://cran.r-project.org/web/packages/GOplot/index.html). *p* <0.05 was considered as statistically significancy.

### Building Protein-Protein Interaction Network

The protein-protein interaction (PPI) network was obtained from the STRING database (https://string-db.org/). By setting the minimum required interaction score >0.7 and hiding the nodes where the network was disconnected, the genes of the hub module were imported into the STRING database to build the PPT network. Cytoscape (http://cytoscape.org/) was used to reconstruct the network. The CytoHubba plug-in in Cytoscape (v3.8.2) can be used to discover critical targets and subnetworks in complex networks. It provided biological data on various biological types, including PPI networks, gene regulation, cellular pathways and signal transduction, as well as help us find the central elements in the network. Eventually, the top 30 gene nodes were ranked by CytoHubba screening.

### Identifying and Validating the Hub Genes

Based on the results of WGCNA and PPI network analysis, Venn analysis (http://bioinformatics.psb.ugent.be/webtools/Venn/) was obtained for the candidate hub genes screened by WGCNA analysis and central nodes screening by CytoHubba. These hub genes obtained by intersection were identified in the immune-related database. Tumour Immune Estimation Resource (TIMER, https://cistrome.shinyapps.io/timer/) was a comprehensive resource for the systematic analysis of the infiltration of the immune cells 10,897 samples based on the 32 cancer types obtained from TCGA (Li et al., 2017). Spearman correlations between the infiltration number of CD4^+^ T cells and the expression of hub genes were calculated, and the results were compared using the R package “ggstatplot” (Patil 2021).

### Constructing the Risk Prediction Model

The testing dataset GSE87371 was used to establish the TME risk module about activated memory CD4^+^ T cells infiltration. The least absolute shrinkage and selection operator (LASSO) Cox regression analysis (Tibshirani 1996) was performed using the R package “glmnet” (https://cran.r-project.org/web/packages/glmnet/) to establish a predictive risk formula. Risk score = *Coefficient1*∗*Expression*1+ *Coefficient* 2∗ *Expression*2+ *Coefficient* 3∗ *Expression*3+……+ *Coefficient*N ∗ *Expression*N. *Coefficient* was the LASSO Cox regression analysis of the hub genes, and *Expression* was the corresponding expression value. All patients were divided into high-risk and low-risk groups separately, and individualized risk scores were calculated using median risk as to the cutoff. Kaplan-Meier survival analysis and log-rank test were used to evaluate the difference in Overall Survival (OS) between high-risk and low-risk groups. Time-dependent receiver operating characteristic (ROC) curves were plotted to evaluate predictive value (Heagerty et al., 2000). The results were mapped using the R packages “ggrisk” (https://cran.r-project.org/web/packages/ggrisk/), “timeROC” (https:/cran.r-project.org/web/packages/timeROC/) and “survival” (https://cran.r-project.org/web/packages/survival/).

### Validating the Risk Prediction Model by the External Validation Sets and Nomogram

The GSE181063, GSE53786, GSE10846, and GSE32918 datasets were downloaded from the GEO databases. The risk score for each enrolled patient was calculated using the same model based on genetic characteristics. Next, the ROC curve and Kaplan-Meier curve are used to test the predicated values of genetic traits. A nomogram integrating clinical characters and the prediction risk model was established based on the GSE87371 cohort to assess the probability of 1-, 3-, and 5-year individualized OS via the “rms” R package (http://cran.r-project,org/web/packages/rms/) (Heagerty et al., 2000). In addition, the discriminatory ability of the nomogram was graphically evaluated by a calibration map.

### Confirming the Expression of Hub Genes in Normal Cases Based on External Validation Sets

The “limma” R package was used to screen for differentially expressed genes (DEGs) in the validation datasets, setting the thresholds (*p*-value <0.05 and |log fold change (FC)| >1) to filter DEGs between normal cases and DLBCL tumor samples. All gene lists sorted by logFC in each dataset were maintained for subsequent integration analysis. Then we used the above result for joint analysis of multiple datasets by using the “RobustRankAggreg” R package (RRA, https://cran.r-project.org/web/packages/RobustRankAggreg/) (Kolde et al., 2012), which is a tool that integrates differential expression analysis results from different platforms, mainly with the RobustRank Aggregation (RRA) algorithm to obtain a comprehensive ranking list. “pheatmap” (https://cran.r-project.org/web/packages/pheatmap/index.html) and “ggplot2” R packages (https://cran.r-project.org/web/packages/ggplot2/index.html) were used to show the heat map and volcano map.

### Carrying out Gene Set Enrichment Analysis (GSEA) and the Finial Hub Gene Mutational Information

Gene set enrichment analysis (GSEA) uses the predefined gene set, sorts the gene according to the degree of the differential expression in the two types of samples, and then checks whether the preset gene set is enriched at the top or bottom of the list. GSEA detects the expression changes of gene sets rather than a single gene (Subramanian et al., 2005). According to the median value of gene expression, the samples were divided into two groups, “c5. go.v7.4. symbols.gmt [Gene ontology]” and “c2. cp.kegg.v7.4. symbols.gmt [Curated]” gene set enrichment analyzes were carried out, with *p*-value <0.05 and *q*-value <0.05 as indicative of statistical significance. The enrichment pathways were visualized according to the protocol (http://www.gsea-msigdb.org/gsea). To further identify the single gene mutation and copy number variation, we studied the TCGA-DLBC using cBioPortal (https://www.cbioportal.org/) for Cancer Genomics.

### Real-Time Quantitative PCR (RT-qPCR)

63 paraffin samples from 2021.12 to 2022.2 from the Department of Pathology of West China Hospital of Sichuan University were screened, of which 42 cases were confirmed as DLBCL samples and 21 samples of normal lymphoid tissue hyperplasia. The Ethical Committee of West China Hospital approved this study and waived informed consent. According to the manufacturer’s protocol, total RNA was extracted from FFPE samples and gDNA removed using the RNApure FFPE kit (CW0535, CoWin Bioscience, Beijing, China). HiScript® III All-in-one RT SuperMix was used Perfect for qPCR (R333, Vazyme, NanJing, China) reverse transcription and used cDNA as a template for real-time fluorescence quantification. RT-qPCR was performed with the SYBR® Green Premix Ex Taq™ II (Tli RNaseH Plus) (RR820A, TaKaRa, Beijing, China) on a Real-time PCR Detection System (Bio-rad). Independent experiments are performed in triplicate, *ß* actin as an internal control. The following primers (Tsingke Biotechnology Co., Ltd., Beijing, China) were used: FCER1G:

F 5′-TCTTCTTTGGCTTCTGGTTCTTC-3′

R 5′-GGGTTCTCCCTTCCCATATTTTA-3′

ACTIN:

F 5′-CCGCGAGAAGATGACCCAGA-3′

R 5′-GATAGCACAGCCTGGATAGCA-3′

## Results

### Overview of the Transcriptomes of and Identification of the Immune Infiltration of DLBCL

**FIGURE 1:**
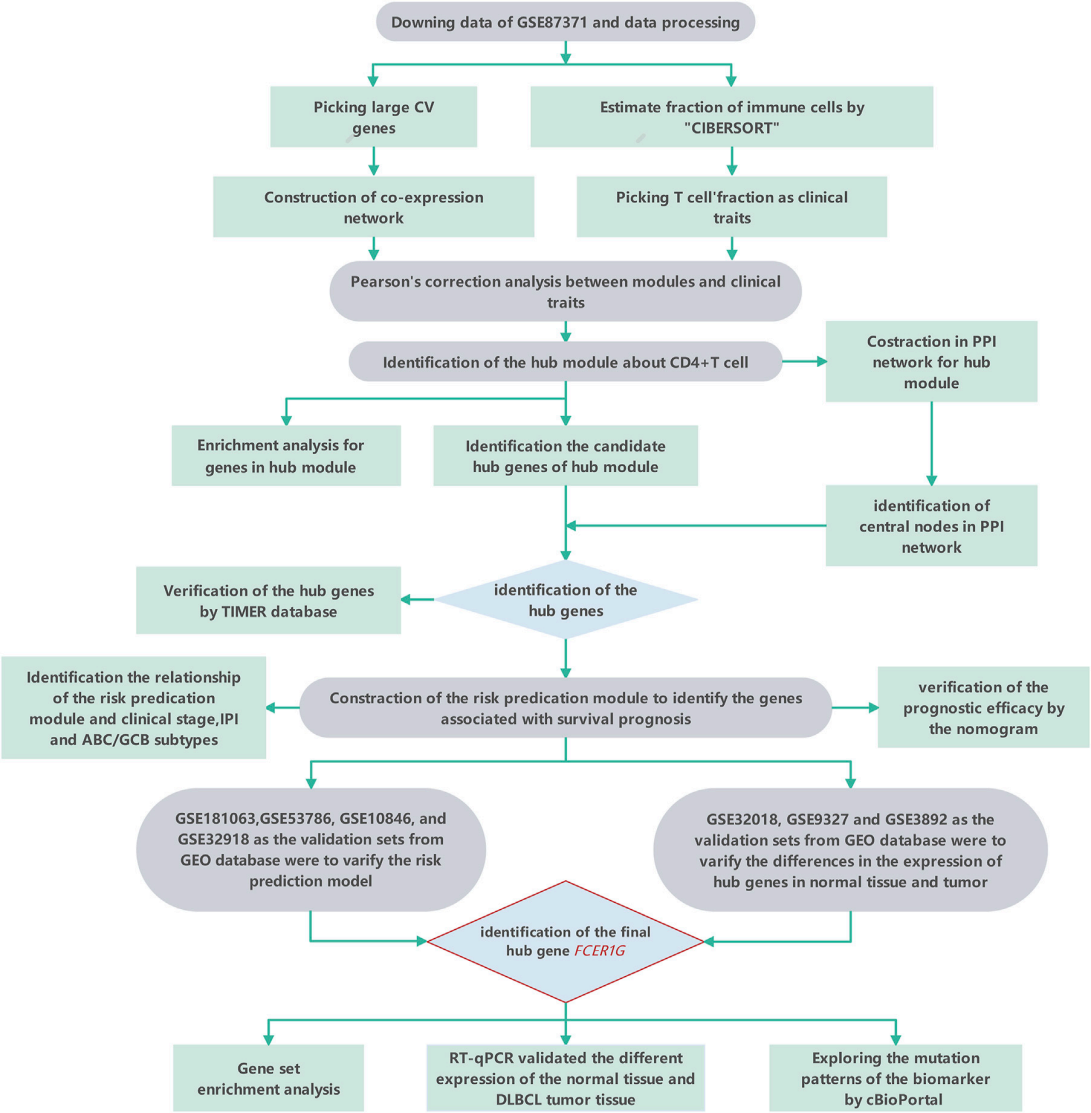
The workflow of the study.

**FIGURE 2:**
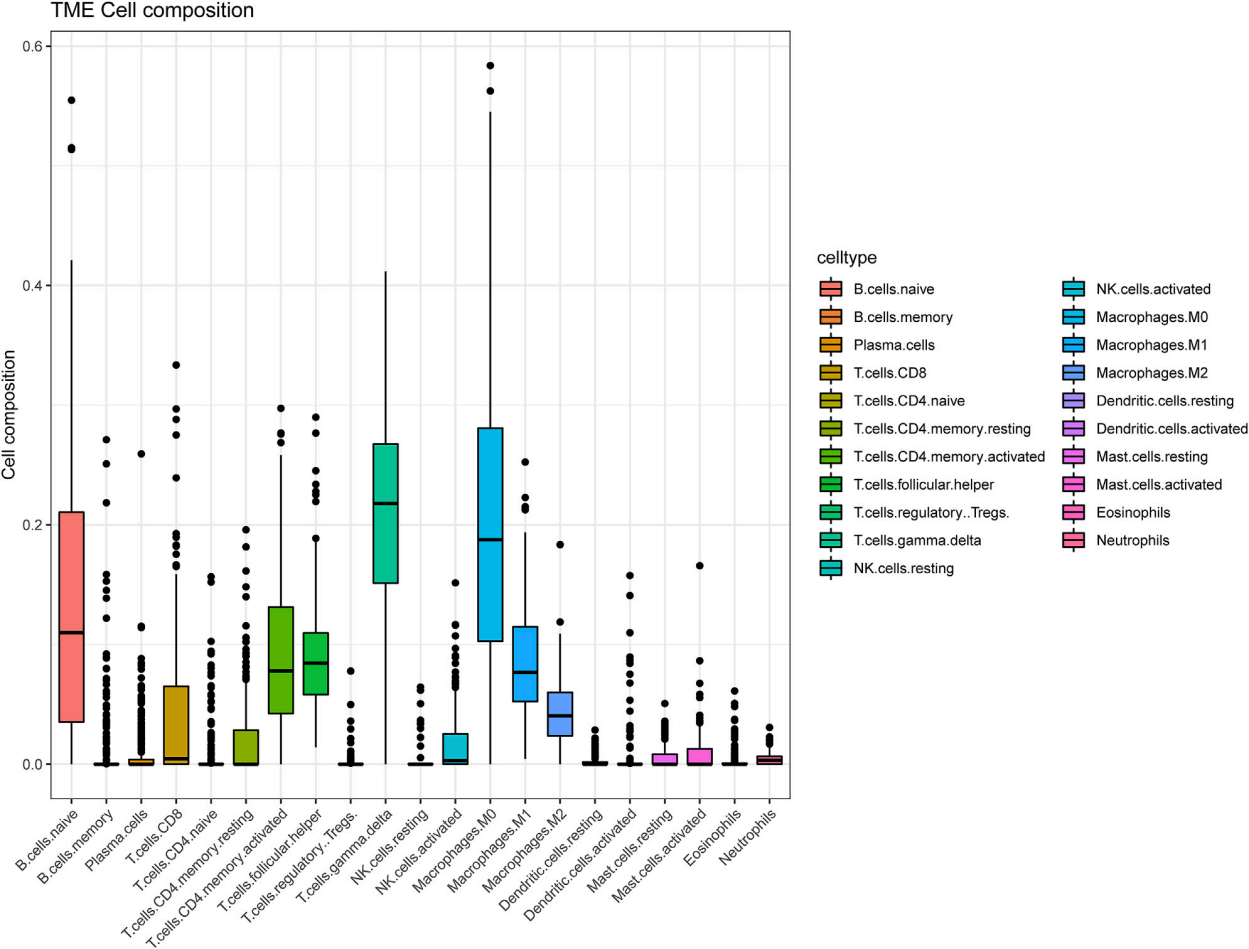
The landscape of immune cells infiltration of DLBCL patients in GSE87371.

The tactics of research was presented in Figure 1. We obtained RNA expression data from 223 DLBCL samples from Gene Expression Omnibus (GEO) database. 12,284 genes were incorporated in the analysis after the expression profile downloading, normalization, standardization, and gene annotation. 7,463 genes were screened out by Coefficient of Variation (CV) >0.1. The RNA expression profile was analyzed using the “CIBERSORT” R package to assess each sample’s abundance of different cell subtypes. The results showed that gamma delta T cells accounted for the most common immune cells in DLBCL (21.2%), followed by M0 phase macrophages (19.3%), and activated memory CD4^+^ T cells accounted for 9.4% (Figure 2, Supplementary Table S1).

### Gene Co-expression Network of DLBCL

**FIGURE 3:**
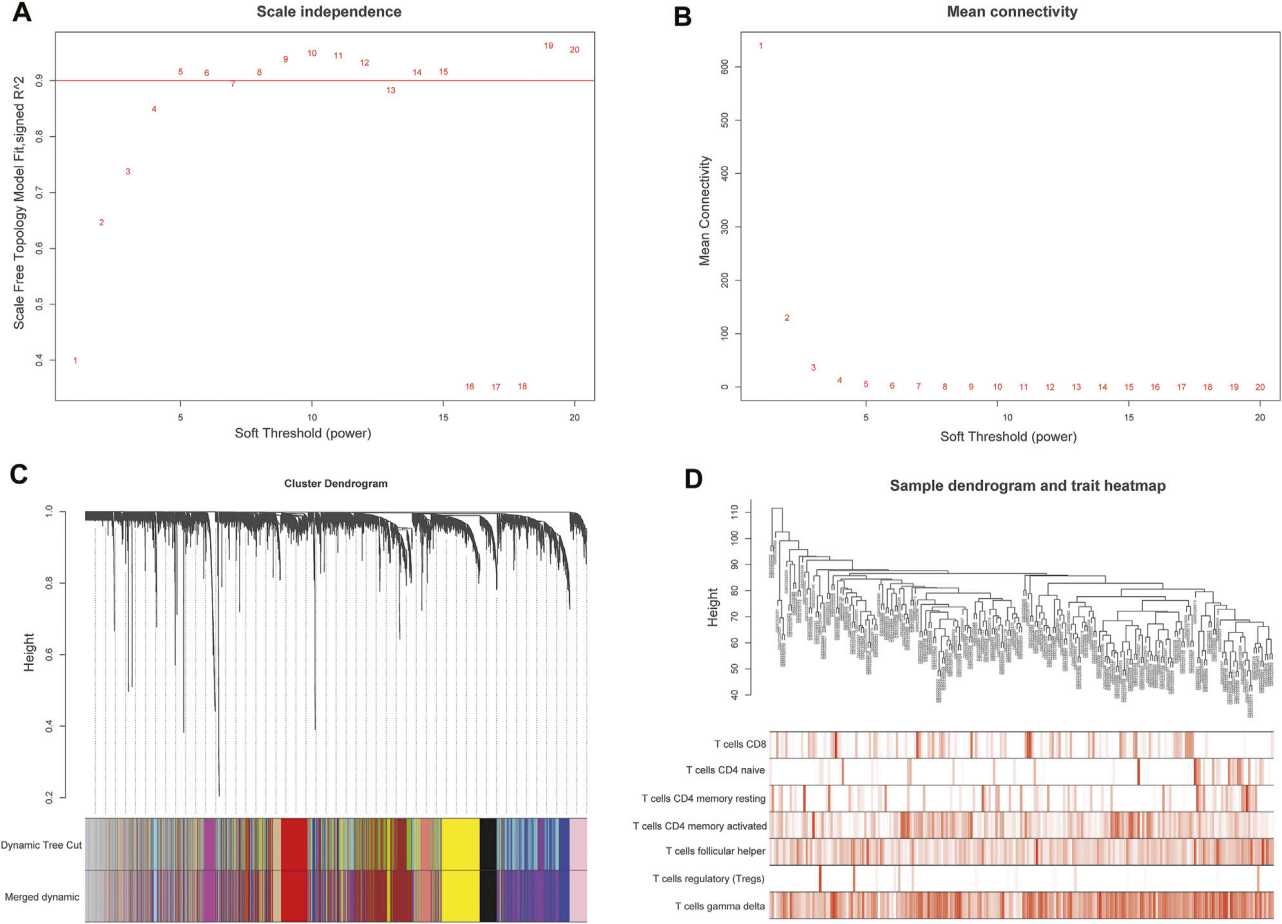
Weighted gene co-expression network analysis (WGCNA) of genes in DLBCL about activated memory CD4^+^ T cells infiltration. **(A)** Analysis of the scale-free fit various soft thresholding power (β). **(B)** Analysis of the average connectivity of 1–20 soft threshold power. **(C)** Hierarchical cluster tree showing co-expression modules identified by WGCNA. **(D)** Sample dendrogram and trait heatmap between the samples and T cells infiltration phenotype.

A weighted co-expression network was constructed by the expression values of 7,463 genes using the “WGCNA” R package. 5,490 genes with the top 75% of the median absolute deviation (MAD) were screened out. at least the MAD was >0.01. The screening principle soft threshold made up the constructed network more in line with the scale-free network characteristics. The soft threshold was set as *ß* = 5 (Figures 3A,B). Hierarchical clustering analysis was carried out based on weighted correlation, and the clustering results were segmented according to the set standards to obtain different gene modules, which were represented by branches and different colors of the cluster tree. The results showed that 15 gene modules were calculated by a hierarchical dynamic tree-cutting algorithm. The number and color of genes represented by each module were black (317 genes), blue (718 genes), cyan (60 genes), green (427genes), green-yellow (118 genes), grey (569 genes), magenta (141 genes), pink (203 genes), purple (138 genes), red (365 genes), salmon (115genes), tan (116 genes), turquoise (807 genes), yellow (678 genes) (Figure 3C). The relationship between module eigengenes was shown in (Supplementary Figure S1A,B). The samples dendrogram and trait heatmap were illustrated to clarify the relationship between samples and T cells phenotypes (Figure 3D).

### Identification of the Hub Module of DLBCL About Activated Memory CD4^+^ T Cells Infiltration and Enrichment Analysis

**FIGURE 4:**
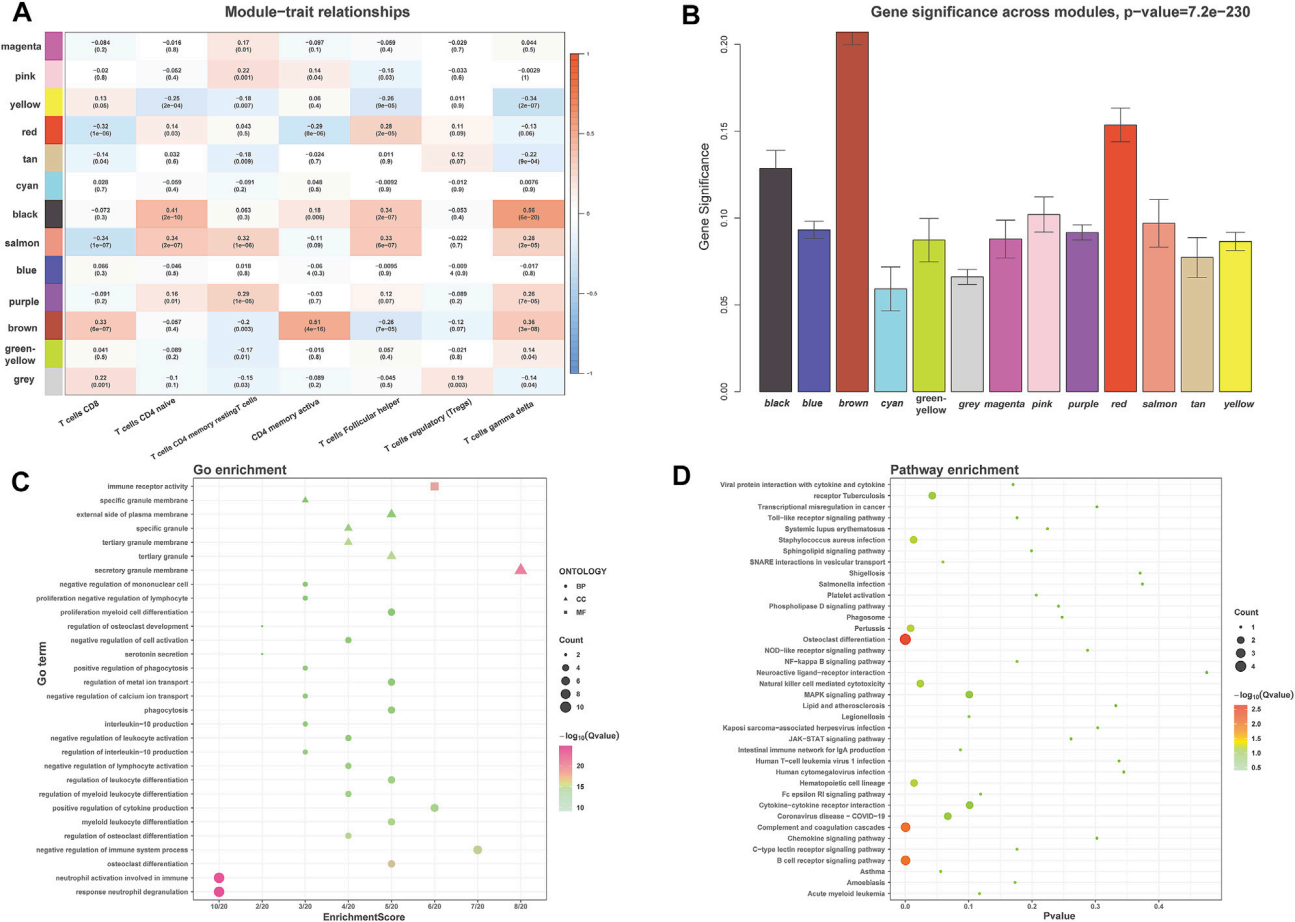
WGCNA identified the significant module about activated memory CD4^+^ T cells infiltration. **(A)** Heatmap showed the correlation of module eigengenes with T cells infiltration. The row represented the module, and the column portrayed the character. The values in the box represented correlation and *p*-value. **(B)** The histogram showed the relationship between different gene modules and gene significance, and the brown module showed the highest significance. **(C,D)** GO **(C)** and KEGG **(D)** analysis for brown module-related genes. GO, Gene Ontology; KEGG, Kyoto Encyclopedia of Genes and Genomes.

The module related to the specific trait was found according to the gene correlation and *p*-value of trait and model eigenvector. Among the fifteen gene modules, the brown module was highly correlated to activated memory CD4^+^ T cells (*R* ^*2*^ = 0.51, *P* = 4e-16) and gamma delta T cells (*R* ^*2*^ = 0.36, *P* = 3e-08). *R* ^*2*^ stands for correlation, and the larger the *R* ^*2*^, the stronger the correlation. Both correlation value and *p*-value showed that the brown gene module had the highest correlation with activated memory CD4^+^ T cells (Figures 4A,B). Therefore, the brown gene module was selected for analysis, which contains 1,115 genes. Enrichment analysis showed that these genes were related to neutrophil activation involved in immune response and immune receptor activity (Figure 4C). KEGG pathway analysis also indicated that the genes were mainly involved in the B cell receptor signaling pathway and osteoclast differentiation, complement and coagulation cascades (Figure 4D).

### Confirmation of the Hub Genes of DLBCL

**FIGURE 5:**
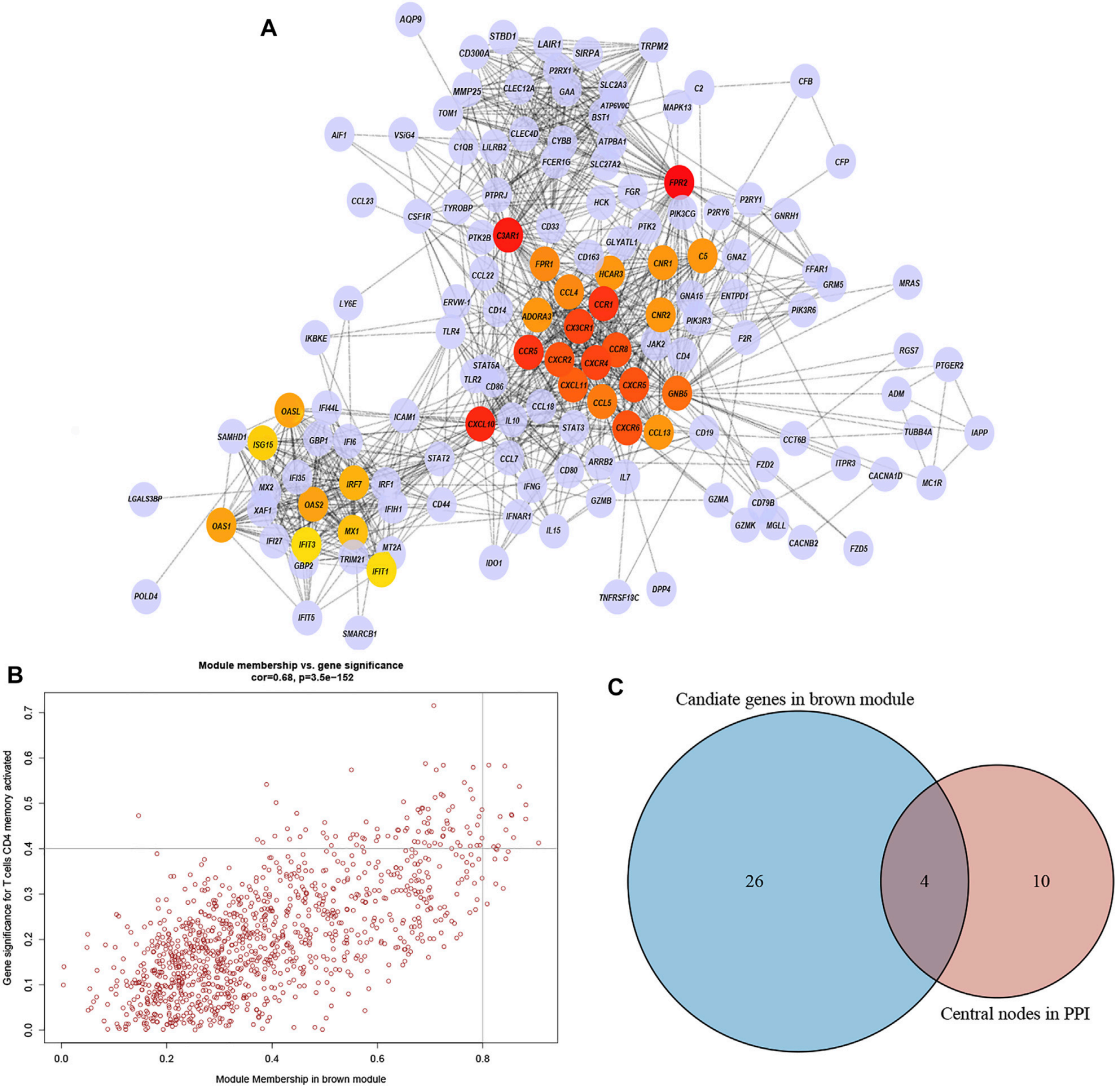
PPI network and identification of the hub genes. **(A)** PPI network from the brown module. The higher the number of connected nodes, the deeper the color of the nodes. **(B)** A scatter plot of the genes in the brown module. Each brown node represented a gene, and dots within the top right corner indicate Gene Significance >0.4 and Module Membership >0.8. **(C)** Hub genes were selected based on the overlap between PPI essential nodes and candidate genes of WGCNA.

**FIGURE 6:**
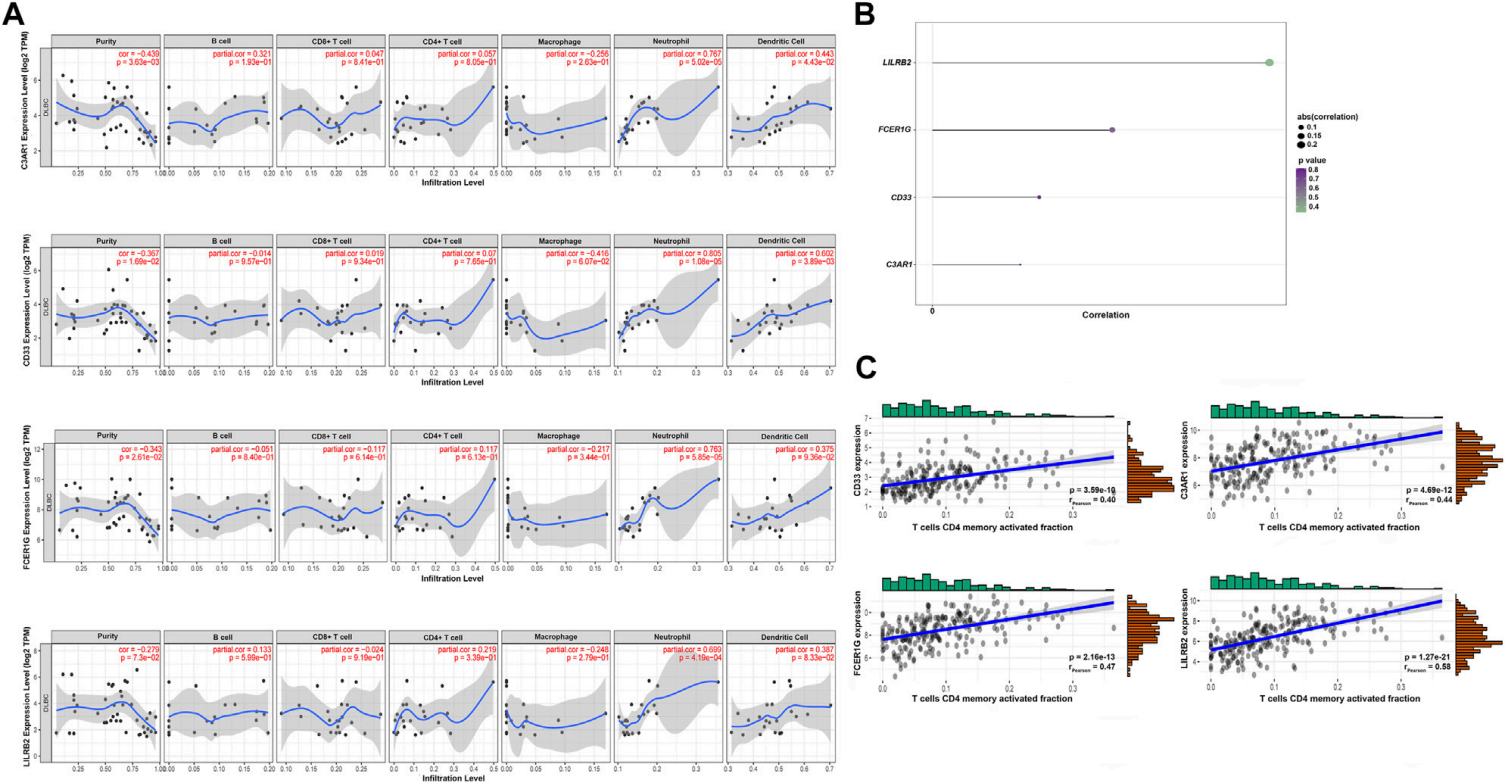
Validation of the hub genes. **(A)** TIMER database showed the relationship between four genes expression and CD4^+^ T cells infiltration. **(B)** Lollipop indicated the relationship between four hub genes expression and activated memory CD4^+^ T cells infiltration degree. *p*-value <0.05 is considered statistically significant. **(C)** Scatter plot of four hub genes expression and activated memory CD4^+^ T cells infiltration degree.

The highly connected genes of the module were investigated as potential vital factors related to activated memory CD4^+^ T cells infiltration number. According to the cut-off standard (Gene-Significance >0.4, Module-Membership >0.8), 14 genes were selected as candidate hub genes (Figure 5B, Supplementary Table S2). The CytoHubba plug-in screened the first 30 gene nodes, and the results were visualized using Cytoscape in the protein-protein network analysis (Figure 5A). Four hub genes (*CD33*, *C3AR1*, *FCER1G*, *LILRB2*) were screened out by the intersection of the two analysis results (Figure 5C). The gene expression profile data in the TIMER database were analyzed to verify the relationship between hub genes and activated memory CD4^+^ T cells (Figure 6A). The results showed a positive correlation of the expression values of the above four genes with the infiltration levels of CD4^+^ T cells (Figures 6B,C).

### Construction and Validation of the Risk Prediction Model Based on the TME of Activated Memory CD4^+^ T Cells Infiltration

**FIGURE 7:**
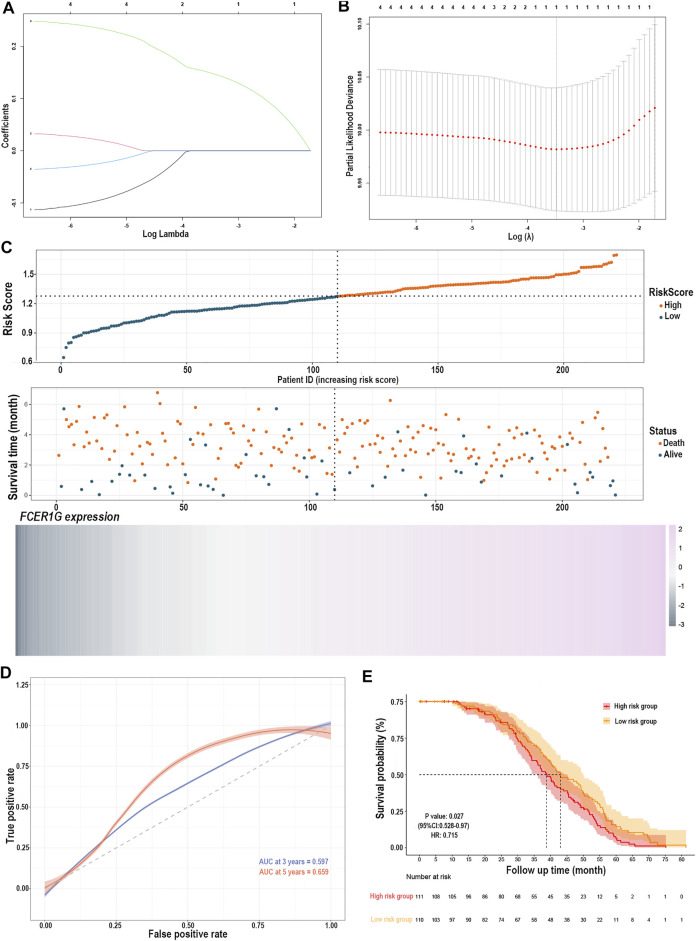
Construction of the risk prediction model. **(A)** The path change chart of the regression coefficient. **(B)** The change curve of penalty term. **(C)** The distribution of risk scores, the survival status of patients, and the expression level in screening single gene. **(D)** The time-dependent ROC curve and AUC of the single-gene signature. **(E)** Kaplan-Meier plots of overall survival between high- and low-risk groups in the testing group by the log-rank test. LASSO, least absolute shrinkage and selection operator; ROC, receiver operating characteristic curve; AUC, an area under the curve.

The LASSO Cox regression analysis identified the correlation between the four gene (*CD33*, *C3AR1*, *FCER1G*, *LILRB2*) expression and overall survival (OS), and determined the optimally weighted coefficient for the predictive activated memory CD4^+^ T cells infiltration-related genes according to variable selection and regularization characteristics. By setting one standard error of the best penalty parameter *λ* value and 1000-fold cross-validation, the path change graph of the regression coefficient was obtained (Figure 7A). The trend of each curve in the figure represented the change of the regression coefficient path. The regression coefficients mainly were compressed to zero, which showed that the module had a good advantage in dimensionality reduction and variable selection. A single-gene predictive signature (*FCER1G*) was obtained from the four hub genes. The left line indicated the optimal value by λ.min criteria (Figure 7B). Then, coefficient values were extracted, and the coefficient of the single gene were multiplied by their mRNA expression levels to calculate individual risk scores using the following formula: Risk score = the mRNA expression level of *FCER1G*∗(0.1485714). Patients from the training group were divided into high-risk and low-risk groups based on the median risk score (Suplementary Table S3). The distributions of the risk scores, survival status of high-risk and low-risk patients, and single-gene prediction model expression levels in the testing dataset are presented in Figure 7C. Time-dependent ROC curve analysis showed that during 3- and 5-year follow-up, the area under the curve (AUC) values were 0.597 and 0.659 (Figure 7D). Survival analysis showed that patients in the high-risk group had significantly shorter median OS than low-risk (Figure 7E). The univariate Cox regression results of the four genes showed that the *p*-value of all three genes was less than 0.05 (Supplementary Figure S2), and the genes most relevant to the survival were screened with LASSO regression. Meantime, the selection of four external validation sets in the GEO database also proved that the single-gene predictive signature (*FCER1G*) had lower expression levels in the low-risk group and higher expression levels in the high-risk group (Supplementary Figures S3A,B,G,H). The AUC values and *p*-value of the Kaplan-Meier survival curve reached statistical significance, and there was the same trend as the testing dataset (Supplementary Figures S3C–F,I–L).

### Validation of the risk Prediction Model by the Nomogram Consisting of a Variety of Clinicopathological Factors

**FIGURE 8:**
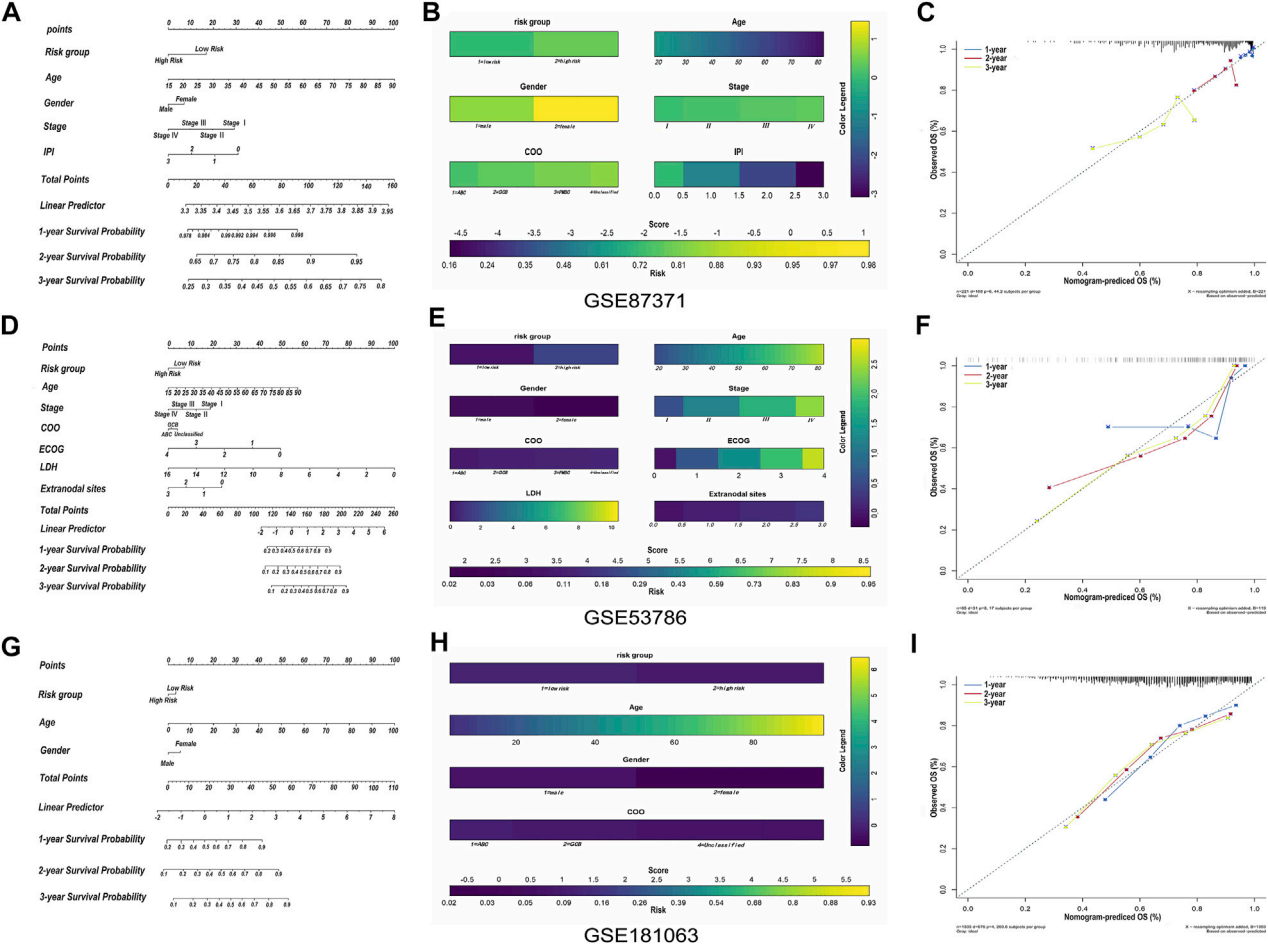
The construction and validation of nomogram. **(A)** Prognostic nomogram for predicting the survival of GSE87371 DLBCL patients and every prediction factor relevant to total score. Clinically, the corresponding score can be obtained according to every patient’s condition, and the total score corresponds to every patient’s correlated survival probability. **(B)** Color nomogram, color legend on the right corresponds to the score of different variables of every GSE87371 DLBCL patient. The scores of multiple variables are added to the bottom total score, with corresponding survival probability. **(C)** Calibration curves of the nomogram predicts the GSE87371 patents’ survival probability at 1-, 2- and 3-year. If the actual curve is closer to the ideal curve, the nomogram predication accuracy is higher. **(D–F)** Nomogram and calibration diagram of validation set GSE53786. **(G–I)** Nomogram and calibration diagram of validation set GSE181063.

The relationship between the single-gene prediction model (*FCER1G*) and other clinical parameters such as the pathological subtypes, clinical stage, and IPI score was performed to understand further by survival analysis. The survival status of patients at different stages showed that the low-risk group had a favorable median OS than the high-risk group (Supplementary Figures S4A,B). Similar results were obtained in the low-risk group with a favorable OS compared with the high-risk patients in IPI <2 and the IPI ≥2 groups (Supplementary Figures S4C,D). Moreover, an analogous results demonstrated that low-risk patients had significantly favorable OS compared to high-risk patients with the activated B-cell-like (ABC) subtype of DLBCL (*p* = 0.0052, HR = 0.42, 95% CI = 0.22–0.77; Supplementary Figure S4E). The same tendency was presented in germinal center B-cell-like (GCB) patients (*p* = 0.0565, HR = 0.63, 95% CI = 0.39–1.01; Supplementary Figure S4F). It was illustrated that the risk prediction model possessed the independent forecasting ability. In addition, a nomogram was established to forecast 1-,2- and 3-year survival based on the clinical pathology factors. The nomograms were developed by assigning each independent predictive factor an initial graphical score, ranging from 0 to 100. The scores for all variables were then summarized to obtain the total score. A vertical line indicated the estimated probability of survival for each DLBCL patient (Figures 8A,B). The calibration chart was attracted to validate the nomogram, which showed an agreement between the predicted and actual survival rates (Figure 8C). ln addition, the validation sets, GSE53786 and GSE181063, both proved that the risk predication model had better prediction performance for 1-, 2-, and 3-year survival conditions (Figures 8D–I).

### Exploring the Molecular Function, Mutation Information and Differential Expression of the Final Key Gene

**FIGURE 9:**
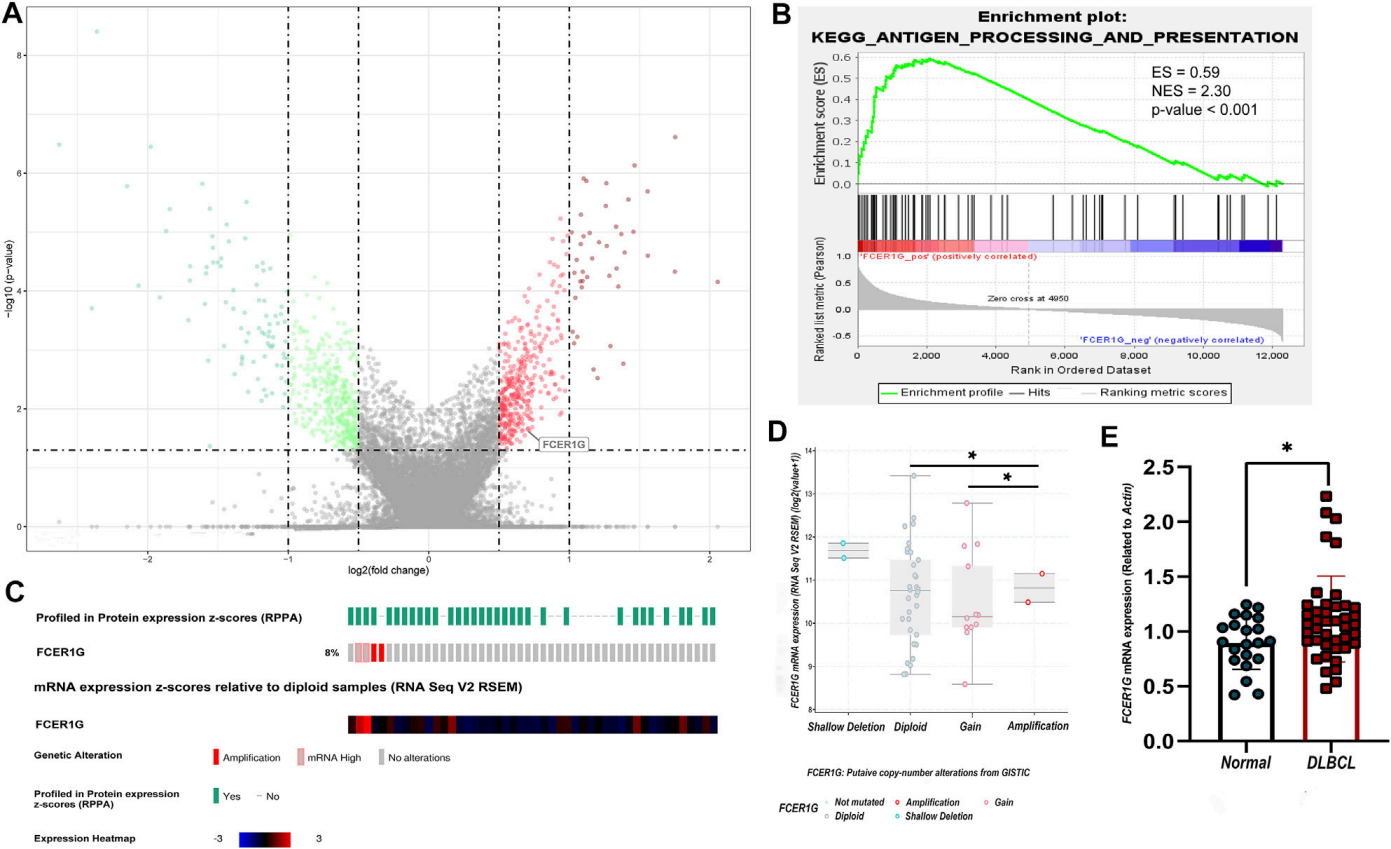
The expression, pathway enrichment and mutation landscape of the final hub gene **(A)** The volcano plot with differentially expressed genes. Red dots indicated overexpression genes, green dots exhibited the low expression genes, and the grey boxed represented meaningless expression genes. **(B)** KEGG pathway analysis of the positive regulation of FCER1G. **(C)** The heatmap of FCER1G mRNA expression and the genetic alteration in the TCGA-DLBC dataset. **(D)** Comparison of FCER1G expression between different types of copy number variation groups. **(E)** Validation of mRNA expression of the final key gene *FCER1G* related with CD4^+^ T cells infiltration degree in FFPE samples (*p*-value = 0.0228).

According to the screening criteria of *p*-value <0.05 and absolute |log fold change (FC)| >1, 124 DEGs were screened between DLBCL samples and normal tissue in the three validation groups GSE32018, GSE9327, and GSE3892 by the algorithm of RobustRank Aggregation (RRA). *FCER1G* were statistically significantly up-regulated in three datasets (Figure 9A). Subsequently, the potential of *FCER1G* biological functions was explored by gene set enrichment analysis (GSEA). KEGG pathway analysis revealed that the high levels of *FCER1G* was most strongly associated with the antigen processing and presentation (Figure 9B; Supplementary Table S4). In addition, according to the TCGA-DLBC samples with complete mRNA and sequencing data (*n* = 48), we assessed the correlation between the *FCER1G* expression and copy number variation in DLBCL. As shown in Figures 9C,D, the copy number variation around the heterotopic point had significantly higher *FCER1G* expression levels. To verify this results in the FFPE samples, RT-qPCR was employed. The expression levels of *FCER1G* in DLBCL tissue and normal lymphoid tissue hyperplasia was significant difference (Figure 9E; *p* = 0.0228). The wet experiment further verified the reliability of bioinformatics results.

## Discussion

Diffuse large B-cell lymphoma (DLBCL) is the most common non-Hodgkin lymphoma subtype and represents a morphologically, biologically, and clinically heterogeneous group of malignant diseases (Pinnix et al., 2016). Recent breakthroughs in immunotherapy have shown a new strategy for the effective treatment of tumors. Many clinical studies aim to improve the overall survival of patients through a unique combination of immunotherapy and chemotherapy (Hude et al., 2017). In addition to the current pathological and clinical predictive factors, reliable and robust biomarkers need to be explored to improve personalized treatment for patients. Thus, immunological markers should be strongly considered in the evaluation and treatment of cancer patients.

As a new therapeutic strategy, treatment aiming at the tumor microenvironment (TME) is a research hotspot. The tumor microenvironment in tumors consists of extracellular matrix as well as the associated stromal cells including immune cells, fibroblasts, and vascular network (Binnewies et al., 2018). Previous studies have focused on the role of cytolytic CD8^+^ T cells as tumor-infiltrating lymphocytes (List et al., 1993). However, recent studies have proved that CD4^+^ T cells may play a central role in the antitumor immune response (Tveita et al., 2015). A subset of CD4^+^ T cells, such as T-regulatory cells (Tregs), mainly infiltrated in the tumor microenvironment, is considered to be pivotal mediators of peripheral tolerance and immune suppression (Facciabene et al., 2012). Therefore, identifying the biomarkers related to CD4^+^ T cells infiltration will facilitate the monitoring of DLBCL immunotherapy response and the exploration of immune infiltration mechanism.

Tumor-infiltrating T lymphocytes (TILs) are considered to play essential roles in the anticancer immune mechanism of the tumor-bearing host in some human solid cancers (Hiraoka et al., 2006). CD4^+^ T cells play a central role in orchestrating the immune response to cancer. Essentially, CD4^+^ T cells recognize peptides represented on MHC class Ⅱ molecules expressed primarily on antigen-presenting cells. In NSCLC, Hiraoka et al. found that the synergistic effect of simultaneous high CD4^+^ T cells and CD8^+^ T cells infiltration in the tumor stroma was a favorable prognostic factor (Hiraoka et al., 2006). A previous study showed that tumor-infiltrating activated CD4^+^ T cells are associated with a good prognosis in head and neck squamous cell carcinoma (Badoual et al., 2006). Some studies have systematically analyzed tumor-infiltrating immune cells in pancreatic ductal carcinoma (PDC) and evaluated their clinicopathological impact. They found that tumor-infiltrating CD4^+^ T^high^ cell was an independent prognosticator helpful in evaluating the immune microenvironment of PDC (Ino et al., 2013). CD4^+^ T cells are some of the essential non-neoplastic immune cells that affect the survival of DLBCL patients and play a vital role in immune monitoring and influencing lymphoma outcome. Some studies have shown that an increase in CD4 cells in the tumor microenvironment before treatment predicts a better prognosis (Judd et al., 2017).

Previous studies have demonstrated that CD4^+^ T cells infiltration of the diseased nodes is a potential predictive indicator of overall survival (OS) and event-free survival (EFS) in DLBCL patients receiving R-CHOP (Keane et al., 2013). The successful application of immune checkpoint inhibitors in DLBCL has increased interest in exploring the potential target of specific immune-related factors for immunotherapy (Xu-Monette et al., 2019). High CD4^+^ T cells enrichment are associated with improved outcomes in many malignancies (Fridman et al., 2012).

Although there have been researches reporting that the presence of increased numbers of activated CD4^+^ T cells in the area of DLBCL predicts a better prognosis (Ansell et al., 2001), the mechanism is unclear. This study identified four hub genes whose expression correlated to CD4^+^ T cell infiltration level, which prompted a possible mechanism to promote tumorigenesis and tumor progression. Among the screened four hub genes, we determined *FCER1G* as a potential predictive biomarker by LASSO regression algorithm and combined analysis of multiple datasets.

Up to now, there are many methods to evaluate the prognosis of DLBCL, which is a malignant tumor that originated from B cells divided into the germinal center cell (GCB) and activated B cell (ABC) (Rosenwald and Staudt, 2003). Cell of origin (COO) is considered to be closely related to the pathogenesis of diseases and has prognostic value (Morin et al., 2021). The most widely used prognostic system of DLBCL in clinical practice is the IPI prognostic scoring system (Sehn et al., 2006). The emergence of high-throughput technologies such as whole exon sequencing and deep sequencing has found a variety of molecular mutations and single nucleotide polymorphisms including *MYD88*, *EZH2*, *CARD11*, *FOX O 1*, involving the abnormalities of multiple signaling pathways including BCR, NK-κB, NOTCH, Toll-like receptors and PI3K, which makes the more in-depth understanding of the pathogenesis and disease susceptibility of DLBCL. Further studies found that *FOX O 1, MYD88* and *EZH2* abnormalities may be associated with the prognosis of DLBCL (Morin et al., 2016). Among other molecular indicators, mutations of *CD5*, *CD30* and *TP53* were relatively studied (Zhang et al., 1999). This study identified four hub genes (*CD33*, *C3AR1*, *FCER1G*, *LILRB2*) whose expression correlated to activated memory CD4^+^ T cells infiltration level, which prompted a possible mechanism to promote tumorigenesis and development. *FCER1G* was identified as a potential predictive biomarker and target by the LASSO Cox regression algorithm and expression in tumor tissues among the screened four hub genes.

Fc Fragment of IgE Receptor Ig (*FCER1G*) mediates allergic inflammatory signaling as a component of the high-affinity immunoglobulin E (IgE) receptor, and it is a critical molecule in developing eczema, clear cell renal cell carcinoma, meningioma and childhood leukemia (Han et al., 2010; Mahachie John et al., 2010; Rajaraman et al., 2010; Chen et al., 2017). Houshi Xu et al. demonstrated *FCER1G* as a novel predictor for clinical diagnosis, prognosis, and response to immunotherapy in glioma patients (Xu et al., 2021). Early studies found that *FCER1G* transduced activation signals from various immunoreceptors and engaged in many immune responses, playing a tumor-promoting role in many kinds of tumors (Shah et al., 2017; Sweet et al., 2017). It was also reported that the demethylation of *FCER1G* was induced by IL15 in the NKp30 + CD8^+^ T cells population exhibiting high natural killer-like anti-tumor potential (Correia et al., 2018). Lin Fu et al. illustrated that the enhanced expression of *FCER1G* predicted a favorable prognosis in multiple myeloma (Fu et al., 2020). Wei Yuan et al. found that *FCER1G* was associated with infiltration of immune cells in the immune microenvironment in esophageal cancer and was a biomarker associated with prognosis (Yuan et al., 2021). The above studies have focused on the effect of *FCER1G* on tumors, finding that *FCER1G* can significantly promote tumor growth, metastasis, angiogenesis, and immune escape.

Our study demonstrated the relationship between the activated memory CD4^+^ T cells infiltration and the development of DLBCL. The WGCNA and CIBERSORT algorithms identified potential biomarkers related to activated memory CD4^+^ T cells in DLBCL. A new risk prediction model for the survival of DLBCL patients was constructed. Eventually, bioinformatics analysis proved that *FCER1G* was identified as potential biomarkers and targets for DLBCL immunotherapy. However, this study has some unavoidable limitations. Further functional research is warranted to explore the molecular functions of the identified genes during DLBCL progression. Ulterior samples are needed to verify these results and the specific mechanism of *FCER1G* in DLBCL requires further investigation.

## Data Availability

The original contributions presented in the study are included in the article/Supplementary Material, further inquiries can be directed to the corresponding author.
