# Supplementary material for: Identification of FCER1G related to Activated Memory CD4+ T Cells Infiltration by Gene Co-expression Network and Construction of a Risk Prediction Module in Diffuse Large B-Cell Lymphoma
Source: Front Genet. 2022 May 30;13:849422. doi: 10.3389/fgene.2022.849422 (PMC9196638; doi:10.3389/fgene.2022.849422)
Supplement: Supplementary file 1 [file DataSheet1.docx]

Supplementary Material

Supplementary material Table S1. Identification of immune-infiltrating levels about DLBCL by CIBERSORT.

| **Sample ID** | **B cells naive** | **B cells memory** | **Plasma cells** | **T cells CD8** | **T cells CD4 naive** | **T cells CD4 memory resting** | **T cells CD4 memory activated** | **T cells follicular helper** | **T cells regulatory (Tregs)** | **T cells gamma delta** | **NK cells resting** | **NK cells activated** | **Monocytes** | **Macrophages M0** | **Macrophages M1** | **Macrophages M2** | **Dendritic cells resting** | **Dendritic cells activated** | **Mast cells resting** | **Mast cells activated** | **Eosinophils** | **Neutrophils** |
| --- | --- | --- | --- | --- | --- | --- | --- | --- | --- | --- | --- | --- | --- | --- | --- | --- | --- | --- | --- | --- | --- | --- |
| **GSM2328918** | 0.3679 | 0.0000 | 0.0007 | 0.0000 | 0.0000 | 0.0248 | 0.0196 | 0.1268 | 0.0000 | 0.1552 | 0.0000 | 0.0148 | 0.0000 | 0.1822 | 0.0270 | 0.0610 | 0.0000 | 0.0000 | 0.0162 | 0.0000 | 0.0000 | 0.0038 |
| **GSM2328919** | 0.0237 | 0.0000 | 0.0167 | 0.1827 | 0.0083 | 0.0000 | 0.1758 | 0.0507 | 0.0000 | 0.1604 | 0.0000 | 0.0455 | 0.0000 | 0.0424 | 0.0520 | 0.0955 | 0.0035 | 0.0867 | 0.0132 | 0.0000 | 0.0270 | 0.0160 |
| **GSM2328920** | 0.0813 | 0.0118 | 0.0000 | 0.0000 | 0.0571 | 0.0000 | 0.0397 | 0.1132 | 0.0000 | 0.3303 | 0.0000 | 0.0175 | 0.0000 | 0.1465 | 0.0836 | 0.0913 | 0.0000 | 0.0000 | 0.0000 | 0.0194 | 0.0000 | 0.0082 |
| **GSM2328921** | 0.2056 | 0.0000 | 0.0000 | 0.0000 | 0.0000 | 0.1959 | 0.0784 | 0.1161 | 0.0000 | 0.2284 | 0.0000 | 0.0000 | 0.0000 | 0.1246 | 0.0180 | 0.0271 | 0.0000 | 0.0000 | 0.0032 | 0.0000 | 0.0000 | 0.0027 |
| **GSM2328922** | 0.1043 | 0.0000 | 0.0000 | 0.0420 | 0.0000 | 0.0616 | 0.0500 | 0.0574 | 0.0000 | 0.2232 | 0.0000 | 0.0000 | 0.0000 | 0.2819 | 0.0593 | 0.0295 | 0.0000 | 0.0000 | 0.0000 | 0.0864 | 0.0000 | 0.0044 |
| **GSM2328923** | 0.1731 | 0.0000 | 0.0000 | 0.0000 | 0.0000 | 0.0651 | 0.0454 | 0.1002 | 0.0179 | 0.1430 | 0.0000 | 0.0000 | 0.0000 | 0.3105 | 0.0680 | 0.0564 | 0.0000 | 0.0000 | 0.0146 | 0.0000 | 0.0000 | 0.0059 |
| **GSM2328924** | 0.0760 | 0.0000 | 0.0422 | 0.0221 | 0.0000 | 0.0000 | 0.0000 | 0.0999 | 0.0000 | 0.3286 | 0.0000 | 0.0474 | 0.0000 | 0.2617 | 0.0788 | 0.0280 | 0.0000 | 0.0000 | 0.0000 | 0.0124 | 0.0000 | 0.0030 |
| **GSM2328925** | 0.0000 | 0.0000 | 0.0373 | 0.1896 | 0.0000 | 0.0000 | 0.1382 | 0.0722 | 0.0000 | 0.2536 | 0.0000 | 0.0908 | 0.0000 | 0.0607 | 0.0751 | 0.0463 | 0.0131 | 0.0000 | 0.0000 | 0.0054 | 0.0000 | 0.0176 |
| **GSM2328926** | 0.0986 | 0.0000 | 0.0000 | 0.0000 | 0.0000 | 0.0209 | 0.0266 | 0.1020 | 0.0000 | 0.3972 | 0.0000 | 0.0000 | 0.0000 | 0.1751 | 0.0786 | 0.0752 | 0.0000 | 0.0000 | 0.0000 | 0.0172 | 0.0015 | 0.0070 |
| **GSM2328927** | 0.0146 | 0.0000 | 0.0035 | 0.0000 | 0.1566 | 0.0000 | 0.2687 | 0.0856 | 0.0000 | 0.1450 | 0.0617 | 0.0000 | 0.0000 | 0.1293 | 0.1124 | 0.0183 | 0.0011 | 0.0000 | 0.0031 | 0.0000 | 0.0000 | 0.0000 |
| **GSM2328928** | 0.1527 | 0.0000 | 0.0571 | 0.0000 | 0.0921 | 0.0293 | 0.0589 | 0.1014 | 0.0000 | 0.2327 | 0.0000 | 0.0000 | 0.0000 | 0.1295 | 0.0584 | 0.0614 | 0.0000 | 0.0000 | 0.0000 | 0.0198 | 0.0003 | 0.0065 |
| **GSM2328929** | 0.1284 | 0.1529 | 0.0000 | 0.0156 | 0.0000 | 0.0370 | 0.0000 | 0.1763 | 0.0000 | 0.1547 | 0.0000 | 0.0898 | 0.0000 | 0.0120 | 0.0894 | 0.1017 | 0.0151 | 0.0000 | 0.0000 | 0.0002 | 0.0269 | 0.0000 |
| **GSM2328930** | 0.3311 | 0.0000 | 0.0226 | 0.0090 | 0.0458 | 0.0000 | 0.1936 | 0.0239 | 0.0000 | 0.1857 | 0.0000 | 0.0000 | 0.0000 | 0.0837 | 0.0133 | 0.0412 | 0.0000 | 0.0224 | 0.0185 | 0.0000 | 0.0037 | 0.0053 |
| **GSM2328931** | 0.0975 | 0.0035 | 0.0000 | 0.0000 | 0.0355 | 0.1059 | 0.0610 | 0.1887 | 0.0000 | 0.2325 | 0.0000 | 0.0000 | 0.0000 | 0.1442 | 0.0894 | 0.0217 | 0.0000 | 0.0000 | 0.0151 | 0.0000 | 0.0000 | 0.0050 |
| **GSM2328932** | 0.0557 | 0.0000 | 0.0000 | 0.0102 | 0.0000 | 0.0000 | 0.0528 | 0.1289 | 0.0000 | 0.2389 | 0.0000 | 0.0383 | 0.0000 | 0.3300 | 0.0993 | 0.0322 | 0.0000 | 0.0000 | 0.0099 | 0.0000 | 0.0039 | 0.0000 |
| **GSM2328933** | 0.0069 | 0.0306 | 0.0132 | 0.1011 | 0.0000 | 0.0000 | 0.1554 | 0.0566 | 0.0000 | 0.3542 | 0.0000 | 0.0058 | 0.0000 | 0.0714 | 0.0673 | 0.1029 | 0.0000 | 0.0274 | 0.0000 | 0.0073 | 0.0000 | 0.0000 |
| **GSM2328934** | 0.0760 | 0.0000 | 0.0000 | 0.0701 | 0.0000 | 0.0000 | 0.0364 | 0.0806 | 0.0000 | 0.2785 | 0.0000 | 0.0040 | 0.0000 | 0.2989 | 0.1379 | 0.0019 | 0.0000 | 0.0000 | 0.0149 | 0.0000 | 0.0008 | 0.0000 |
| **GSM2328935** | 0.0538 | 0.0000 | 0.0000 | 0.0035 | 0.0000 | 0.0398 | 0.0832 | 0.1253 | 0.0000 | 0.2546 | 0.0000 | 0.0000 | 0.0000 | 0.2476 | 0.1236 | 0.0586 | 0.0000 | 0.0000 | 0.0000 | 0.0073 | 0.0000 | 0.0028 |
| **GSM2328936** | 0.0256 | 0.0418 | 0.0000 | 0.0681 | 0.0000 | 0.0000 | 0.0825 | 0.0937 | 0.0000 | 0.0908 | 0.0000 | 0.0228 | 0.0000 | 0.5128 | 0.0482 | 0.0105 | 0.0000 | 0.0000 | 0.0000 | 0.0031 | 0.0000 | 0.0000 |
| **GSM2328937** | 0.0690 | 0.0000 | 0.0571 | 0.0000 | 0.0766 | 0.0299 | 0.1297 | 0.0923 | 0.0000 | 0.3653 | 0.0000 | 0.0000 | 0.0000 | 0.1035 | 0.0348 | 0.0282 | 0.0000 | 0.0000 | 0.0000 | 0.0000 | 0.0000 | 0.0136 |
| **GSM2328938** | 0.2114 | 0.0000 | 0.0000 | 0.0000 | 0.0000 | 0.0738 | 0.0614 | 0.0609 | 0.0000 | 0.1575 | 0.0000 | 0.0079 | 0.0000 | 0.2250 | 0.1379 | 0.0408 | 0.0065 | 0.0000 | 0.0000 | 0.0000 | 0.0000 | 0.0169 |
| **GSM2328939** | 0.2229 | 0.0000 | 0.0000 | 0.0000 | 0.0000 | 0.0000 | 0.0580 | 0.0988 | 0.0294 | 0.1241 | 0.0000 | 0.0000 | 0.0000 | 0.1909 | 0.1257 | 0.1090 | 0.0127 | 0.0000 | 0.0000 | 0.0108 | 0.0064 | 0.0112 |
| **GSM2328940** | 0.1526 | 0.0000 | 0.0000 | 0.0000 | 0.0353 | 0.0000 | 0.0434 | 0.2452 | 0.0043 | 0.2228 | 0.0000 | 0.0000 | 0.0000 | 0.1969 | 0.0529 | 0.0433 | 0.0000 | 0.0000 | 0.0000 | 0.0016 | 0.0000 | 0.0016 |
| **GSM2328941** | 0.2952 | 0.0000 | 0.0000 | 0.0000 | 0.0350 | 0.0000 | 0.0488 | 0.2767 | 0.0000 | 0.1611 | 0.0000 | 0.0000 | 0.0000 | 0.1251 | 0.0050 | 0.0432 | 0.0000 | 0.0000 | 0.0092 | 0.0000 | 0.0000 | 0.0007 |
| **GSM2328942** | 0.1071 | 0.0000 | 0.0041 | 0.0000 | 0.0639 | 0.0467 | 0.0534 | 0.1136 | 0.0000 | 0.2229 | 0.0000 | 0.0000 | 0.0000 | 0.2743 | 0.0599 | 0.0369 | 0.0000 | 0.0000 | 0.0172 | 0.0000 | 0.0000 | 0.0000 |
| **GSM2328943** | 0.1274 | 0.0000 | 0.0000 | 0.0000 | 0.0470 | 0.1059 | 0.0722 | 0.1575 | 0.0000 | 0.2865 | 0.0000 | 0.0000 | 0.0000 | 0.1159 | 0.0352 | 0.0440 | 0.0000 | 0.0000 | 0.0068 | 0.0000 | 0.0000 | 0.0015 |
| **GSM2328944** | 0.2036 | 0.0000 | 0.0000 | 0.0751 | 0.0000 | 0.0000 | 0.0668 | 0.1010 | 0.0000 | 0.1031 | 0.0000 | 0.0400 | 0.0000 | 0.3083 | 0.0169 | 0.0547 | 0.0000 | 0.0136 | 0.0000 | 0.0009 | 0.0161 | 0.0000 |
| **GSM2328945** | 0.0026 | 0.0000 | 0.0000 | 0.0167 | 0.0000 | 0.0000 | 0.1355 | 0.1648 | 0.0033 | 0.1939 | 0.0000 | 0.0000 | 0.0000 | 0.3629 | 0.1000 | 0.0000 | 0.0033 | 0.0000 | 0.0169 | 0.0000 | 0.0000 | 0.0000 |
| **GSM2328946** | 0.2203 | 0.0000 | 0.0000 | 0.0000 | 0.0000 | 0.0271 | 0.0065 | 0.1607 | 0.0000 | 0.3182 | 0.0000 | 0.0253 | 0.0000 | 0.0674 | 0.0991 | 0.0539 | 0.0044 | 0.0000 | 0.0000 | 0.0170 | 0.0000 | 0.0000 |
| **GSM2328947** | 0.0307 | 0.0000 | 0.0000 | 0.0350 | 0.0000 | 0.0000 | 0.0751 | 0.0914 | 0.0000 | 0.3032 | 0.0000 | 0.0277 | 0.0000 | 0.3199 | 0.0573 | 0.0133 | 0.0000 | 0.0321 | 0.0000 | 0.0104 | 0.0000 | 0.0039 |
| **GSM2328948** | 0.0232 | 0.0000 | 0.0000 | 0.1527 | 0.0000 | 0.0000 | 0.0387 | 0.0820 | 0.0029 | 0.1848 | 0.0000 | 0.0199 | 0.0000 | 0.2349 | 0.1158 | 0.0034 | 0.0000 | 0.1099 | 0.0252 | 0.0000 | 0.0000 | 0.0065 |
| **GSM2328949** | 0.0000 | 0.0000 | 0.0508 | 0.0000 | 0.0000 | 0.0000 | 0.0711 | 0.1105 | 0.0000 | 0.2926 | 0.0000 | 0.1072 | 0.0000 | 0.0469 | 0.0565 | 0.1084 | 0.0096 | 0.0842 | 0.0000 | 0.0099 | 0.0294 | 0.0228 |
| **GSM2328950** | 0.3463 | 0.0000 | 0.0401 | 0.0000 | 0.0000 | 0.0000 | 0.0160 | 0.1100 | 0.0000 | 0.1654 | 0.0000 | 0.0000 | 0.0000 | 0.1705 | 0.0456 | 0.0987 | 0.0000 | 0.0000 | 0.0074 | 0.0000 | 0.0000 | 0.0000 |
| **GSM2328951** | 0.2409 | 0.0000 | 0.0000 | 0.0000 | 0.0000 | 0.0438 | 0.0324 | 0.0819 | 0.0000 | 0.2376 | 0.0000 | 0.0642 | 0.0000 | 0.1023 | 0.1008 | 0.0877 | 0.0000 | 0.0000 | 0.0000 | 0.0030 | 0.0000 | 0.0053 |
| **GSM2328952** | 0.1284 | 0.1387 | 0.0000 | 0.0000 | 0.0138 | 0.0432 | 0.0042 | 0.1412 | 0.0000 | 0.2276 | 0.0000 | 0.0060 | 0.0000 | 0.1666 | 0.0716 | 0.0318 | 0.0000 | 0.0000 | 0.0000 | 0.0218 | 0.0000 | 0.0051 |
| **GSM2328953** | 0.0658 | 0.0000 | 0.0171 | 0.1589 | 0.0000 | 0.0000 | 0.1764 | 0.0733 | 0.0000 | 0.3132 | 0.0000 | 0.0550 | 0.0000 | 0.0000 | 0.0509 | 0.0390 | 0.0157 | 0.0000 | 0.0000 | 0.0215 | 0.0024 | 0.0108 |
| **GSM2328954** | 0.0088 | 0.0359 | 0.0547 | 0.0000 | 0.0000 | 0.0408 | 0.0856 | 0.1089 | 0.0000 | 0.2173 | 0.0000 | 0.0000 | 0.0000 | 0.3101 | 0.0923 | 0.0312 | 0.0000 | 0.0000 | 0.0110 | 0.0000 | 0.0000 | 0.0033 |
| **GSM2328955** | 0.1376 | 0.0000 | 0.0000 | 0.0092 | 0.0000 | 0.0000 | 0.1329 | 0.1225 | 0.0000 | 0.1332 | 0.0000 | 0.0724 | 0.0000 | 0.1399 | 0.1431 | 0.0650 | 0.0102 | 0.0000 | 0.0000 | 0.0219 | 0.0000 | 0.0121 |
| **GSM2328956** | 0.0242 | 0.0000 | 0.0000 | 0.3336 | 0.0000 | 0.0000 | 0.0393 | 0.1094 | 0.0000 | 0.3153 | 0.0000 | 0.0000 | 0.0000 | 0.0000 | 0.0712 | 0.0600 | 0.0173 | 0.0000 | 0.0000 | 0.0202 | 0.0052 | 0.0043 |
| **GSM2328957** | 0.0876 | 0.0000 | 0.0266 | 0.0000 | 0.0828 | 0.1615 | 0.0947 | 0.0411 | 0.0000 | 0.2640 | 0.0000 | 0.0000 | 0.0000 | 0.0011 | 0.1385 | 0.0726 | 0.0000 | 0.0000 | 0.0000 | 0.0066 | 0.0212 | 0.0016 |
| **GSM2328958** | 0.0411 | 0.0000 | 0.0883 | 0.0592 | 0.0022 | 0.0000 | 0.0945 | 0.0941 | 0.0000 | 0.3393 | 0.0000 | 0.0000 | 0.0000 | 0.0923 | 0.1313 | 0.0343 | 0.0000 | 0.0000 | 0.0000 | 0.0191 | 0.0000 | 0.0042 |
| **GSM2328959** | 0.0000 | 0.0341 | 0.0657 | 0.0719 | 0.0000 | 0.0000 | 0.1272 | 0.0332 | 0.0000 | 0.2162 | 0.0000 | 0.0000 | 0.0000 | 0.2662 | 0.0924 | 0.0888 | 0.0000 | 0.0000 | 0.0000 | 0.0010 | 0.0000 | 0.0032 |
| **GSM2328960** | 0.0780 | 0.0000 | 0.0000 | 0.0000 | 0.0332 | 0.0098 | 0.1628 | 0.1037 | 0.0000 | 0.2698 | 0.0000 | 0.0000 | 0.0000 | 0.2027 | 0.1090 | 0.0278 | 0.0000 | 0.0000 | 0.0031 | 0.0000 | 0.0000 | 0.0000 |
| **GSM2328961** | 0.0607 | 0.0035 | 0.0000 | 0.0663 | 0.0000 | 0.0000 | 0.0312 | 0.1164 | 0.0000 | 0.1871 | 0.0000 | 0.0000 | 0.0000 | 0.3250 | 0.1865 | 0.0062 | 0.0000 | 0.0000 | 0.0171 | 0.0000 | 0.0000 | 0.0000 |
| **GSM2328962** | 0.2422 | 0.0000 | 0.0000 | 0.0254 | 0.0000 | 0.0000 | 0.0521 | 0.0341 | 0.0000 | 0.1139 | 0.0000 | 0.0000 | 0.0000 | 0.4110 | 0.0962 | 0.0000 | 0.0000 | 0.0000 | 0.0212 | 0.0000 | 0.0000 | 0.0037 |
| **GSM2328963** | 0.0736 | 0.0000 | 0.0000 | 0.0000 | 0.0000 | 0.0000 | 0.0214 | 0.0737 | 0.0008 | 0.1017 | 0.0000 | 0.0000 | 0.0000 | 0.4413 | 0.2524 | 0.0000 | 0.0000 | 0.0000 | 0.0351 | 0.0000 | 0.0000 | 0.0000 |
| **GSM2328964** | 0.0000 | 0.0000 | 0.0451 | 0.1494 | 0.0000 | 0.0000 | 0.2313 | 0.0411 | 0.0000 | 0.2256 | 0.0000 | 0.1167 | 0.0000 | 0.0132 | 0.1074 | 0.0415 | 0.0078 | 0.0000 | 0.0000 | 0.0000 | 0.0063 | 0.0146 |
| **GSM2328965** | 0.1593 | 0.0000 | 0.0000 | 0.0220 | 0.0000 | 0.0922 | 0.0885 | 0.1244 | 0.0000 | 0.3039 | 0.0000 | 0.0000 | 0.0000 | 0.1333 | 0.0518 | 0.0168 | 0.0000 | 0.0000 | 0.0064 | 0.0000 | 0.0000 | 0.0015 |
| **GSM2328966** | 0.2753 | 0.0000 | 0.0018 | 0.0092 | 0.0000 | 0.0000 | 0.1226 | 0.0460 | 0.0111 | 0.0000 | 0.0000 | 0.0263 | 0.0000 | 0.3858 | 0.0749 | 0.0362 | 0.0045 | 0.0000 | 0.0032 | 0.0000 | 0.0031 | 0.0000 |
| **GSM2328967** | 0.0852 | 0.0000 | 0.0440 | 0.0000 | 0.0000 | 0.0000 | 0.1385 | 0.0789 | 0.0000 | 0.1363 | 0.0000 | 0.0000 | 0.0000 | 0.4037 | 0.0953 | 0.0041 | 0.0000 | 0.0000 | 0.0115 | 0.0000 | 0.0000 | 0.0025 |
| **GSM2328969** | 0.2500 | 0.0000 | 0.0000 | 0.0000 | 0.0000 | 0.0618 | 0.1321 | 0.0711 | 0.0000 | 0.1522 | 0.0000 | 0.0077 | 0.0000 | 0.1868 | 0.0487 | 0.0860 | 0.0000 | 0.0000 | 0.0034 | 0.0000 | 0.0000 | 0.0000 |
| **GSM2328970** | 0.0230 | 0.0000 | 0.0000 | 0.0374 | 0.0000 | 0.0000 | 0.0723 | 0.0601 | 0.0029 | 0.1895 | 0.0000 | 0.0077 | 0.0000 | 0.5185 | 0.0748 | 0.0000 | 0.0000 | 0.0000 | 0.0137 | 0.0000 | 0.0000 | 0.0000 |
| **GSM2328971** | 0.1347 | 0.2711 | 0.0000 | 0.0000 | 0.0254 | 0.0000 | 0.0791 | 0.0196 | 0.0043 | 0.0315 | 0.0299 | 0.0000 | 0.0000 | 0.3212 | 0.0764 | 0.0000 | 0.0000 | 0.0000 | 0.0067 | 0.0000 | 0.0000 | 0.0000 |
| **GSM2328972** | 0.0768 | 0.0000 | 0.0033 | 0.1831 | 0.0000 | 0.0000 | 0.0253 | 0.0797 | 0.0000 | 0.1532 | 0.0000 | 0.0293 | 0.0000 | 0.3556 | 0.0603 | 0.0281 | 0.0000 | 0.0000 | 0.0000 | 0.0051 | 0.0001 | 0.0000 |
| **GSM2328973** | 0.0225 | 0.0000 | 0.0000 | 0.0000 | 0.1522 | 0.0006 | 0.0372 | 0.2279 | 0.0000 | 0.3574 | 0.0000 | 0.0000 | 0.0000 | 0.1379 | 0.0067 | 0.0154 | 0.0000 | 0.0296 | 0.0097 | 0.0000 | 0.0002 | 0.0027 |
| **GSM2328974** | 0.2055 | 0.0000 | 0.0000 | 0.0000 | 0.0000 | 0.0000 | 0.0422 | 0.1308 | 0.0000 | 0.2419 | 0.0000 | 0.0000 | 0.0000 | 0.3175 | 0.0566 | 0.0017 | 0.0000 | 0.0000 | 0.0000 | 0.0038 | 0.0000 | 0.0000 |
| **GSM2328975** | 0.2451 | 0.0000 | 0.0000 | 0.0000 | 0.0212 | 0.0313 | 0.1194 | 0.0587 | 0.0000 | 0.2330 | 0.0000 | 0.0052 | 0.0000 | 0.1898 | 0.0512 | 0.0346 | 0.0000 | 0.0000 | 0.0000 | 0.0078 | 0.0000 | 0.0028 |
| **GSM2328976** | 0.0213 | 0.0030 | 0.0000 | 0.0767 | 0.0000 | 0.0000 | 0.1453 | 0.0692 | 0.0000 | 0.2481 | 0.0000 | 0.0000 | 0.0000 | 0.2595 | 0.1233 | 0.0452 | 0.0045 | 0.0000 | 0.0000 | 0.0000 | 0.0002 | 0.0037 |
| **GSM2328977** | 0.5136 | 0.0000 | 0.0000 | 0.0000 | 0.0026 | 0.0146 | 0.0286 | 0.0857 | 0.0000 | 0.0972 | 0.0645 | 0.0000 | 0.0000 | 0.1040 | 0.0347 | 0.0449 | 0.0000 | 0.0000 | 0.0000 | 0.0079 | 0.0000 | 0.0016 |
| **GSM2328978** | 0.1837 | 0.0000 | 0.0000 | 0.0263 | 0.0000 | 0.0000 | 0.0000 | 0.2338 | 0.0000 | 0.1730 | 0.0000 | 0.0603 | 0.0000 | 0.2561 | 0.0459 | 0.0000 | 0.0033 | 0.0000 | 0.0000 | 0.0177 | 0.0000 | 0.0000 |
| **GSM2328979** | 0.5548 | 0.0000 | 0.0000 | 0.0000 | 0.0000 | 0.1156 | 0.0051 | 0.0298 | 0.0000 | 0.1130 | 0.0000 | 0.0210 | 0.0000 | 0.0199 | 0.0463 | 0.0529 | 0.0000 | 0.0000 | 0.0337 | 0.0000 | 0.0000 | 0.0079 |
| **GSM2328980** | 0.3594 | 0.1585 | 0.0000 | 0.0263 | 0.0000 | 0.0364 | 0.0754 | 0.0917 | 0.0000 | 0.0000 | 0.0367 | 0.0000 | 0.0000 | 0.0922 | 0.0519 | 0.0560 | 0.0000 | 0.0000 | 0.0000 | 0.0121 | 0.0000 | 0.0034 |
| **GSM2328981** | 0.1566 | 0.0000 | 0.0000 | 0.0549 | 0.0000 | 0.0000 | 0.1926 | 0.0681 | 0.0000 | 0.3105 | 0.0000 | 0.0732 | 0.0000 | 0.0000 | 0.0670 | 0.0598 | 0.0076 | 0.0000 | 0.0000 | 0.0000 | 0.0098 | 0.0000 |
| **GSM2328982** | 0.0697 | 0.0369 | 0.0000 | 0.0000 | 0.0000 | 0.0651 | 0.1132 | 0.0579 | 0.0000 | 0.2652 | 0.0000 | 0.0000 | 0.0000 | 0.2039 | 0.0753 | 0.0424 | 0.0000 | 0.0007 | 0.0000 | 0.0556 | 0.0000 | 0.0142 |
| **GSM2328983** | 0.2154 | 0.0000 | 0.0000 | 0.0358 | 0.0000 | 0.0000 | 0.0563 | 0.1012 | 0.0000 | 0.2170 | 0.0000 | 0.0054 | 0.0000 | 0.2494 | 0.0888 | 0.0135 | 0.0000 | 0.0000 | 0.0124 | 0.0000 | 0.0000 | 0.0049 |
| **GSM2328984** | 0.1909 | 0.0000 | 0.0000 | 0.0000 | 0.0000 | 0.0000 | 0.0147 | 0.1464 | 0.0000 | 0.2676 | 0.0000 | 0.0463 | 0.0000 | 0.2599 | 0.0523 | 0.0000 | 0.0000 | 0.0000 | 0.0000 | 0.0182 | 0.0000 | 0.0037 |
| **GSM2328985** | 0.0599 | 0.0000 | 0.0000 | 0.0122 | 0.0250 | 0.0000 | 0.1799 | 0.0409 | 0.0000 | 0.1458 | 0.0000 | 0.0000 | 0.0000 | 0.3484 | 0.1147 | 0.0000 | 0.0000 | 0.0236 | 0.0264 | 0.0000 | 0.0231 | 0.0000 |
| **GSM2328986** | 0.0105 | 0.0091 | 0.0207 | 0.0062 | 0.0000 | 0.0000 | 0.2571 | 0.0382 | 0.0000 | 0.3230 | 0.0000 | 0.0000 | 0.0000 | 0.0804 | 0.2128 | 0.0299 | 0.0003 | 0.0000 | 0.0084 | 0.0000 | 0.0000 | 0.0035 |
| **GSM2328987** | 0.0804 | 0.0000 | 0.0000 | 0.0439 | 0.0000 | 0.0000 | 0.1231 | 0.0515 | 0.0000 | 0.2685 | 0.0000 | 0.0024 | 0.0000 | 0.2434 | 0.1248 | 0.0593 | 0.0009 | 0.0000 | 0.0020 | 0.0000 | 0.0000 | 0.0000 |
| **GSM2328988** | 0.2067 | 0.0000 | 0.0000 | 0.0000 | 0.0000 | 0.0708 | 0.0250 | 0.0759 | 0.0000 | 0.1887 | 0.0000 | 0.0000 | 0.0000 | 0.2323 | 0.0660 | 0.1048 | 0.0000 | 0.0000 | 0.0000 | 0.0297 | 0.0000 | 0.0000 |
| **GSM2328989** | 0.1329 | 0.0000 | 0.0346 | 0.0008 | 0.0000 | 0.0000 | 0.1134 | 0.0708 | 0.0000 | 0.1815 | 0.0000 | 0.0056 | 0.0000 | 0.3577 | 0.0656 | 0.0294 | 0.0000 | 0.0000 | 0.0050 | 0.0000 | 0.0000 | 0.0027 |
| **GSM2328990** | 0.0084 | 0.0173 | 0.0000 | 0.0202 | 0.0000 | 0.0000 | 0.0271 | 0.0563 | 0.0058 | 0.0924 | 0.0000 | 0.0044 | 0.0000 | 0.5625 | 0.0514 | 0.0075 | 0.0000 | 0.1409 | 0.0053 | 0.0000 | 0.0006 | 0.0000 |
| **GSM2328991** | 0.3615 | 0.0000 | 0.0443 | 0.0000 | 0.0095 | 0.0112 | 0.0370 | 0.1114 | 0.0000 | 0.1983 | 0.0000 | 0.0127 | 0.0000 | 0.0504 | 0.0350 | 0.1188 | 0.0000 | 0.0000 | 0.0098 | 0.0000 | 0.0000 | 0.0000 |
| **GSM2328992** | 0.3343 | 0.0000 | 0.0000 | 0.0000 | 0.0045 | 0.0000 | 0.0016 | 0.1292 | 0.0000 | 0.2561 | 0.0000 | 0.0000 | 0.0000 | 0.1061 | 0.0542 | 0.0946 | 0.0000 | 0.0000 | 0.0000 | 0.0133 | 0.0024 | 0.0035 |
| **GSM2328993** | 0.3672 | 0.0000 | 0.0122 | 0.0310 | 0.0000 | 0.0000 | 0.0644 | 0.0444 | 0.0000 | 0.0572 | 0.0000 | 0.0000 | 0.0000 | 0.3327 | 0.0309 | 0.0600 | 0.0000 | 0.0000 | 0.0000 | 0.0000 | 0.0000 | 0.0000 |
| **GSM2328994** | 0.0162 | 0.0010 | 0.0000 | 0.0245 | 0.0000 | 0.0000 | 0.2583 | 0.0960 | 0.0000 | 0.2573 | 0.0000 | 0.0000 | 0.0000 | 0.2135 | 0.1126 | 0.0151 | 0.0000 | 0.0000 | 0.0000 | 0.0000 | 0.0000 | 0.0056 |
| **GSM2328995** | 0.0673 | 0.0000 | 0.0825 | 0.0000 | 0.0000 | 0.0000 | 0.1790 | 0.0862 | 0.0000 | 0.3087 | 0.0000 | 0.0349 | 0.0000 | 0.0609 | 0.0781 | 0.0579 | 0.0195 | 0.0000 | 0.0000 | 0.0155 | 0.0000 | 0.0096 |
| **GSM2328996** | 0.1397 | 0.0000 | 0.0309 | 0.0000 | 0.0000 | 0.0094 | 0.0701 | 0.0991 | 0.0000 | 0.3514 | 0.0000 | 0.0000 | 0.0000 | 0.1599 | 0.0888 | 0.0420 | 0.0000 | 0.0000 | 0.0000 | 0.0044 | 0.0000 | 0.0043 |
| **GSM2328997** | 0.0336 | 0.0000 | 0.0000 | 0.1666 | 0.0000 | 0.0000 | 0.1117 | 0.0795 | 0.0000 | 0.2211 | 0.0000 | 0.0330 | 0.0000 | 0.0790 | 0.1070 | 0.0507 | 0.0000 | 0.0895 | 0.0121 | 0.0000 | 0.0049 | 0.0113 |
| **GSM2328998** | 0.0156 | 0.0214 | 0.0528 | 0.0000 | 0.0000 | 0.0145 | 0.0701 | 0.0727 | 0.0000 | 0.2334 | 0.0000 | 0.0397 | 0.0000 | 0.3523 | 0.0402 | 0.0616 | 0.0000 | 0.0000 | 0.0000 | 0.0096 | 0.0056 | 0.0103 |
| **GSM2328999** | 0.0616 | 0.0000 | 0.0000 | 0.0557 | 0.0000 | 0.0000 | 0.2317 | 0.0452 | 0.0000 | 0.2279 | 0.0000 | 0.0124 | 0.0000 | 0.0590 | 0.1055 | 0.0880 | 0.0000 | 0.0000 | 0.0507 | 0.0000 | 0.0481 | 0.0141 |
| **GSM2329000** | 0.0142 | 0.0921 | 0.0000 | 0.0000 | 0.0000 | 0.0289 | 0.0000 | 0.1647 | 0.0000 | 0.2778 | 0.0000 | 0.0229 | 0.0000 | 0.2520 | 0.1107 | 0.0000 | 0.0203 | 0.0000 | 0.0000 | 0.0164 | 0.0000 | 0.0000 |
| **GSM2329001** | 0.1561 | 0.0000 | 0.0000 | 0.0000 | 0.0238 | 0.0000 | 0.0850 | 0.1528 | 0.0000 | 0.3072 | 0.0000 | 0.0000 | 0.0000 | 0.1816 | 0.0573 | 0.0252 | 0.0000 | 0.0050 | 0.0025 | 0.0000 | 0.0000 | 0.0037 |
| **GSM2329002** | 0.1186 | 0.0000 | 0.0000 | 0.0000 | 0.0000 | 0.0520 | 0.1210 | 0.0876 | 0.0000 | 0.2467 | 0.0000 | 0.0000 | 0.0000 | 0.2522 | 0.1094 | 0.0093 | 0.0000 | 0.0000 | 0.0000 | 0.0000 | 0.0000 | 0.0030 |
| **GSM2329003** | 0.1278 | 0.0000 | 0.0000 | 0.0000 | 0.0000 | 0.0000 | 0.0818 | 0.0720 | 0.0000 | 0.2707 | 0.0000 | 0.0000 | 0.0000 | 0.1882 | 0.1865 | 0.0350 | 0.0000 | 0.0000 | 0.0284 | 0.0000 | 0.0000 | 0.0096 |
| **GSM2329004** | 0.0271 | 0.0000 | 0.0000 | 0.0391 | 0.0000 | 0.0000 | 0.0628 | 0.0905 | 0.0000 | 0.2219 | 0.0000 | 0.0218 | 0.0000 | 0.3919 | 0.1292 | 0.0000 | 0.0000 | 0.0000 | 0.0000 | 0.0157 | 0.0000 | 0.0000 |
| **GSM2329005** | 0.0209 | 0.0105 | 0.0127 | 0.0000 | 0.0161 | 0.0000 | 0.1924 | 0.0850 | 0.0000 | 0.2443 | 0.0000 | 0.0070 | 0.0000 | 0.2449 | 0.1148 | 0.0346 | 0.0049 | 0.0000 | 0.0000 | 0.0058 | 0.0000 | 0.0060 |
| **GSM2329006** | 0.0848 | 0.0467 | 0.0000 | 0.0000 | 0.0000 | 0.0000 | 0.1308 | 0.1596 | 0.0000 | 0.1607 | 0.0000 | 0.0000 | 0.0000 | 0.1906 | 0.1326 | 0.0784 | 0.0000 | 0.0000 | 0.0000 | 0.0069 | 0.0000 | 0.0089 |
| **GSM2329007** | 0.2271 | 0.0000 | 0.0000 | 0.0000 | 0.0000 | 0.0000 | 0.0650 | 0.1038 | 0.0000 | 0.2534 | 0.0000 | 0.0268 | 0.0000 | 0.2005 | 0.0782 | 0.0407 | 0.0000 | 0.0000 | 0.0000 | 0.0000 | 0.0000 | 0.0045 |
| **GSM2329008** | 0.3114 | 0.0000 | 0.0622 | 0.0000 | 0.1026 | 0.0355 | 0.0867 | 0.0876 | 0.0000 | 0.1298 | 0.0000 | 0.0000 | 0.0000 | 0.0781 | 0.0355 | 0.0651 | 0.0000 | 0.0000 | 0.0000 | 0.0000 | 0.0007 | 0.0046 |
| **GSM2329009** | 0.0038 | 0.0666 | 0.0000 | 0.1301 | 0.0000 | 0.0000 | 0.1162 | 0.0481 | 0.0000 | 0.2225 | 0.0000 | 0.0000 | 0.0000 | 0.0428 | 0.1867 | 0.0661 | 0.0068 | 0.0443 | 0.0263 | 0.0000 | 0.0365 | 0.0030 |
| **GSM2329010** | 0.1800 | 0.0000 | 0.0000 | 0.0000 | 0.0584 | 0.0000 | 0.0364 | 0.0595 | 0.0000 | 0.1121 | 0.0000 | 0.0000 | 0.0000 | 0.3723 | 0.0777 | 0.0916 | 0.0000 | 0.0000 | 0.0000 | 0.0061 | 0.0000 | 0.0058 |
| **GSM2329011** | 0.0050 | 0.2509 | 0.0508 | 0.0022 | 0.0000 | 0.0000 | 0.0424 | 0.0615 | 0.0034 | 0.0752 | 0.0223 | 0.0000 | 0.0000 | 0.4128 | 0.0044 | 0.0302 | 0.0000 | 0.0135 | 0.0000 | 0.0200 | 0.0000 | 0.0053 |
| **GSM2329012** | 0.0096 | 0.0000 | 0.0000 | 0.1506 | 0.0000 | 0.0000 | 0.0451 | 0.0790 | 0.0000 | 0.1857 | 0.0000 | 0.0411 | 0.0000 | 0.3160 | 0.1411 | 0.0091 | 0.0040 | 0.0000 | 0.0000 | 0.0156 | 0.0031 | 0.0000 |
| **GSM2329013** | 0.0256 | 0.0000 | 0.0000 | 0.1755 | 0.0000 | 0.0000 | 0.1195 | 0.0165 | 0.0000 | 0.2254 | 0.0000 | 0.0000 | 0.0000 | 0.2253 | 0.1415 | 0.0396 | 0.0000 | 0.0000 | 0.0146 | 0.0000 | 0.0120 | 0.0046 |
| **GSM2329014** | 0.0683 | 0.0000 | 0.0000 | 0.0320 | 0.0000 | 0.0000 | 0.1951 | 0.0886 | 0.0000 | 0.2421 | 0.0000 | 0.0223 | 0.0000 | 0.0563 | 0.2228 | 0.0415 | 0.0084 | 0.0000 | 0.0122 | 0.0000 | 0.0000 | 0.0104 |
| **GSM2329015** | 0.2639 | 0.0000 | 0.0000 | 0.0000 | 0.0000 | 0.0000 | 0.0286 | 0.1046 | 0.0070 | 0.0757 | 0.0000 | 0.0406 | 0.0000 | 0.2476 | 0.0757 | 0.0871 | 0.0038 | 0.0000 | 0.0097 | 0.0000 | 0.0508 | 0.0049 |
| **GSM2329016** | 0.3975 | 0.0000 | 0.1153 | 0.0000 | 0.0046 | 0.0380 | 0.0000 | 0.0394 | 0.0000 | 0.0924 | 0.0000 | 0.0043 | 0.0000 | 0.1648 | 0.0288 | 0.0588 | 0.0000 | 0.0000 | 0.0000 | 0.0211 | 0.0288 | 0.0063 |
| **GSM2329017** | 0.3226 | 0.0000 | 0.0198 | 0.0000 | 0.0122 | 0.0000 | 0.0300 | 0.1179 | 0.0360 | 0.2198 | 0.0000 | 0.0259 | 0.0000 | 0.0863 | 0.0279 | 0.0680 | 0.0000 | 0.0000 | 0.0000 | 0.0190 | 0.0095 | 0.0051 |
| **GSM2329019** | 0.3859 | 0.0000 | 0.0000 | 0.0000 | 0.0000 | 0.0577 | 0.0733 | 0.0879 | 0.0000 | 0.1496 | 0.0000 | 0.0034 | 0.0000 | 0.1604 | 0.0589 | 0.0000 | 0.0000 | 0.0000 | 0.0146 | 0.0000 | 0.0030 | 0.0052 |
| **GSM2329020** | 0.0209 | 0.0259 | 0.0000 | 0.0109 | 0.0000 | 0.0224 | 0.0994 | 0.0638 | 0.0000 | 0.3126 | 0.0000 | 0.0000 | 0.0000 | 0.2324 | 0.1441 | 0.0359 | 0.0000 | 0.0000 | 0.0205 | 0.0000 | 0.0000 | 0.0112 |
| **GSM2329021** | 0.0423 | 0.0800 | 0.0000 | 0.0401 | 0.0000 | 0.0000 | 0.0676 | 0.0674 | 0.0000 | 0.2120 | 0.0000 | 0.0000 | 0.0000 | 0.2829 | 0.1933 | 0.0000 | 0.0000 | 0.0000 | 0.0139 | 0.0000 | 0.0005 | 0.0000 |
| **GSM2329022** | 0.2935 | 0.0000 | 0.1141 | 0.0525 | 0.0225 | 0.0362 | 0.0885 | 0.0644 | 0.0000 | 0.1375 | 0.0000 | 0.0770 | 0.0000 | 0.0000 | 0.0679 | 0.0346 | 0.0000 | 0.0000 | 0.0090 | 0.0000 | 0.0000 | 0.0023 |
| **GSM2329023** | 0.2518 | 0.0000 | 0.0000 | 0.0987 | 0.0000 | 0.0000 | 0.0944 | 0.0524 | 0.0000 | 0.1406 | 0.0000 | 0.0842 | 0.0000 | 0.0647 | 0.1470 | 0.0386 | 0.0000 | 0.0000 | 0.0000 | 0.0256 | 0.0000 | 0.0020 |
| **GSM2329024** | 0.0199 | 0.0000 | 0.0000 | 0.1822 | 0.0000 | 0.0000 | 0.0992 | 0.0904 | 0.0000 | 0.2371 | 0.0000 | 0.0687 | 0.0000 | 0.0217 | 0.2141 | 0.0237 | 0.0159 | 0.0000 | 0.0000 | 0.0128 | 0.0066 | 0.0077 |
| **GSM2329026** | 0.0597 | 0.2185 | 0.0000 | 0.0000 | 0.0000 | 0.0683 | 0.0352 | 0.0891 | 0.0000 | 0.1695 | 0.0334 | 0.0000 | 0.0000 | 0.1783 | 0.0514 | 0.0755 | 0.0000 | 0.0000 | 0.0000 | 0.0160 | 0.0000 | 0.0052 |
| **GSM2329027** | 0.0357 | 0.0003 | 0.0000 | 0.1176 | 0.0000 | 0.0000 | 0.1526 | 0.0774 | 0.0000 | 0.2617 | 0.0000 | 0.0162 | 0.0000 | 0.1420 | 0.1226 | 0.0661 | 0.0000 | 0.0000 | 0.0000 | 0.0079 | 0.0000 | 0.0000 |
| **GSM2329029** | 0.0124 | 0.0495 | 0.0000 | 0.0210 | 0.0000 | 0.0000 | 0.0379 | 0.0782 | 0.0000 | 0.2010 | 0.0000 | 0.0000 | 0.0000 | 0.4769 | 0.0956 | 0.0075 | 0.0007 | 0.0000 | 0.0174 | 0.0000 | 0.0020 | 0.0000 |
| **GSM2329030** | 0.3291 | 0.0000 | 0.0000 | 0.0000 | 0.0000 | 0.0000 | 0.0000 | 0.0904 | 0.0000 | 0.1397 | 0.0000 | 0.0284 | 0.0000 | 0.1389 | 0.0326 | 0.0703 | 0.0000 | 0.0000 | 0.0000 | 0.1658 | 0.0000 | 0.0048 |
| **GSM2329031** | 0.2405 | 0.0000 | 0.0000 | 0.1174 | 0.0000 | 0.0000 | 0.0611 | 0.0843 | 0.0000 | 0.1319 | 0.0000 | 0.0279 | 0.0000 | 0.2299 | 0.0626 | 0.0323 | 0.0000 | 0.0000 | 0.0000 | 0.0115 | 0.0000 | 0.0006 |
| **GSM2329032** | 0.0752 | 0.0000 | 0.0000 | 0.1572 | 0.0000 | 0.0000 | 0.2313 | 0.0269 | 0.0000 | 0.2482 | 0.0000 | 0.0065 | 0.0000 | 0.0350 | 0.1652 | 0.0440 | 0.0047 | 0.0000 | 0.0006 | 0.0000 | 0.0000 | 0.0051 |
| **GSM2329033** | 0.1134 | 0.0000 | 0.0000 | 0.0840 | 0.0000 | 0.0000 | 0.1584 | 0.0529 | 0.0000 | 0.2421 | 0.0000 | 0.0000 | 0.0000 | 0.2559 | 0.0477 | 0.0364 | 0.0000 | 0.0000 | 0.0000 | 0.0090 | 0.0000 | 0.0001 |
| **GSM2329034** | 0.0060 | 0.1453 | 0.0000 | 0.0017 | 0.0000 | 0.0000 | 0.2565 | 0.0320 | 0.0000 | 0.2291 | 0.0000 | 0.0041 | 0.0000 | 0.0496 | 0.1937 | 0.0571 | 0.0096 | 0.0000 | 0.0000 | 0.0016 | 0.0131 | 0.0008 |
| **GSM2329035** | 0.2255 | 0.0000 | 0.0424 | 0.0000 | 0.0000 | 0.0371 | 0.0639 | 0.0988 | 0.0000 | 0.2080 | 0.0000 | 0.0891 | 0.0000 | 0.0527 | 0.0911 | 0.0588 | 0.0059 | 0.0000 | 0.0000 | 0.0193 | 0.0025 | 0.0049 |
| **GSM2329037** | 0.2079 | 0.0000 | 0.0000 | 0.1288 | 0.0000 | 0.0000 | 0.0315 | 0.0587 | 0.0000 | 0.1633 | 0.0000 | 0.0033 | 0.0000 | 0.1993 | 0.1349 | 0.0648 | 0.0000 | 0.0000 | 0.0075 | 0.0000 | 0.0000 | 0.0000 |
| **GSM2329038** | 0.1770 | 0.0000 | 0.0794 | 0.1250 | 0.0000 | 0.0000 | 0.1166 | 0.0675 | 0.0000 | 0.1637 | 0.0000 | 0.0617 | 0.0000 | 0.0000 | 0.0709 | 0.0734 | 0.0088 | 0.0000 | 0.0310 | 0.0000 | 0.0092 | 0.0159 |
| **GSM2329039** | 0.2633 | 0.0000 | 0.0000 | 0.0000 | 0.0000 | 0.0853 | 0.0678 | 0.0364 | 0.0000 | 0.1422 | 0.0000 | 0.0000 | 0.0000 | 0.2650 | 0.0523 | 0.0640 | 0.0000 | 0.0000 | 0.0204 | 0.0000 | 0.0006 | 0.0027 |
| **GSM2329040** | 0.0787 | 0.0000 | 0.0000 | 0.0000 | 0.0656 | 0.0000 | 0.1353 | 0.0973 | 0.0000 | 0.2579 | 0.0000 | 0.0968 | 0.0000 | 0.1092 | 0.0544 | 0.0490 | 0.0165 | 0.0000 | 0.0000 | 0.0175 | 0.0121 | 0.0098 |
| **GSM2329041** | 0.0530 | 0.0327 | 0.0000 | 0.0000 | 0.0000 | 0.0000 | 0.0657 | 0.1667 | 0.0215 | 0.2216 | 0.0000 | 0.0135 | 0.0000 | 0.2873 | 0.0202 | 0.0480 | 0.0145 | 0.0287 | 0.0000 | 0.0000 | 0.0155 | 0.0110 |
| **GSM2329042** | 0.0408 | 0.0000 | 0.0000 | 0.0477 | 0.0000 | 0.0771 | 0.1559 | 0.0459 | 0.0000 | 0.2109 | 0.0000 | 0.0000 | 0.0000 | 0.1190 | 0.2125 | 0.0684 | 0.0000 | 0.0000 | 0.0142 | 0.0000 | 0.0000 | 0.0076 |
| **GSM2329043** | 0.0253 | 0.0000 | 0.0015 | 0.2968 | 0.0000 | 0.0000 | 0.1380 | 0.0337 | 0.0000 | 0.3413 | 0.0000 | 0.0503 | 0.0000 | 0.0196 | 0.0359 | 0.0410 | 0.0129 | 0.0000 | 0.0000 | 0.0000 | 0.0000 | 0.0036 |
| **GSM2329044** | 0.2909 | 0.0000 | 0.0000 | 0.0469 | 0.0000 | 0.0000 | 0.1021 | 0.0372 | 0.0000 | 0.2041 | 0.0000 | 0.0000 | 0.0000 | 0.1849 | 0.0762 | 0.0358 | 0.0000 | 0.0000 | 0.0219 | 0.0000 | 0.0000 | 0.0000 |
| **GSM2329045** | 0.1770 | 0.0000 | 0.0122 | 0.1014 | 0.0000 | 0.0000 | 0.0505 | 0.0977 | 0.0000 | 0.1513 | 0.0000 | 0.0399 | 0.0000 | 0.2869 | 0.0535 | 0.0037 | 0.0000 | 0.0000 | 0.0000 | 0.0260 | 0.0000 | 0.0000 |
| **GSM2329046** | 0.1394 | 0.0000 | 0.0294 | 0.2880 | 0.0000 | 0.0000 | 0.1483 | 0.0214 | 0.0000 | 0.2479 | 0.0000 | 0.0129 | 0.0000 | 0.0184 | 0.0695 | 0.0201 | 0.0000 | 0.0000 | 0.0000 | 0.0000 | 0.0000 | 0.0048 |
| **GSM2329047** | 0.1104 | 0.0000 | 0.0247 | 0.0000 | 0.0000 | 0.0000 | 0.1104 | 0.0812 | 0.0000 | 0.3114 | 0.0000 | 0.0283 | 0.0000 | 0.1885 | 0.1025 | 0.0351 | 0.0015 | 0.0000 | 0.0000 | 0.0000 | 0.0000 | 0.0060 |
| **GSM2329048** | 0.3571 | 0.0000 | 0.0642 | 0.0000 | 0.0000 | 0.0507 | 0.0428 | 0.0730 | 0.0000 | 0.1255 | 0.0000 | 0.0170 | 0.0000 | 0.1477 | 0.0771 | 0.0396 | 0.0000 | 0.0000 | 0.0000 | 0.0015 | 0.0000 | 0.0037 |
| **GSM2329049** | 0.1505 | 0.0000 | 0.0000 | 0.0000 | 0.0353 | 0.0440 | 0.1050 | 0.0906 | 0.0000 | 0.3956 | 0.0000 | 0.0000 | 0.0000 | 0.0618 | 0.0349 | 0.0474 | 0.0000 | 0.0000 | 0.0000 | 0.0180 | 0.0080 | 0.0088 |
| **GSM2329050** | 0.1130 | 0.0000 | 0.0721 | 0.0133 | 0.0000 | 0.0000 | 0.1330 | 0.0503 | 0.0000 | 0.0962 | 0.0000 | 0.0697 | 0.0000 | 0.3236 | 0.0601 | 0.0585 | 0.0000 | 0.0000 | 0.0000 | 0.0090 | 0.0000 | 0.0012 |
| **GSM2329051** | 0.0237 | 0.0000 | 0.0083 | 0.1652 | 0.0000 | 0.0000 | 0.0834 | 0.1267 | 0.0000 | 0.1232 | 0.0000 | 0.0623 | 0.0000 | 0.1886 | 0.0672 | 0.0890 | 0.0000 | 0.0000 | 0.0000 | 0.0363 | 0.0086 | 0.0176 |
| **GSM2329052** | 0.0030 | 0.0798 | 0.0000 | 0.0906 | 0.0122 | 0.0000 | 0.0000 | 0.1298 | 0.0090 | 0.2129 | 0.0000 | 0.1515 | 0.0000 | 0.0206 | 0.2152 | 0.0490 | 0.0016 | 0.0000 | 0.0000 | 0.0000 | 0.0250 | 0.0000 |
| **GSM2329053** | 0.1014 | 0.0175 | 0.0000 | 0.0000 | 0.0000 | 0.0032 | 0.0102 | 0.0889 | 0.0000 | 0.0788 | 0.0000 | 0.0119 | 0.0000 | 0.5450 | 0.0950 | 0.0480 | 0.0000 | 0.0000 | 0.0000 | 0.0000 | 0.0000 | 0.0000 |
| **GSM2329054** | 0.0590 | 0.0406 | 0.0000 | 0.0000 | 0.0000 | 0.0000 | 0.0510 | 0.1771 | 0.0498 | 0.1096 | 0.0345 | 0.0000 | 0.0000 | 0.3416 | 0.0804 | 0.0000 | 0.0285 | 0.0000 | 0.0000 | 0.0279 | 0.0000 | 0.0000 |
| **GSM2329055** | 0.3005 | 0.0000 | 0.0000 | 0.0000 | 0.0000 | 0.1055 | 0.1549 | 0.0844 | 0.0000 | 0.0500 | 0.0506 | 0.0000 | 0.0000 | 0.1662 | 0.0453 | 0.0092 | 0.0000 | 0.0000 | 0.0277 | 0.0000 | 0.0024 | 0.0034 |
| **GSM2329056** | 0.0555 | 0.0000 | 0.0337 | 0.0129 | 0.0000 | 0.0003 | 0.2974 | 0.0678 | 0.0000 | 0.2441 | 0.0000 | 0.0000 | 0.0000 | 0.1839 | 0.0722 | 0.0287 | 0.0000 | 0.0000 | 0.0000 | 0.0000 | 0.0000 | 0.0036 |
| **GSM2329057** | 0.1874 | 0.0000 | 0.0000 | 0.0000 | 0.0802 | 0.0929 | 0.1324 | 0.1403 | 0.0000 | 0.1871 | 0.0000 | 0.0000 | 0.0000 | 0.1240 | 0.0224 | 0.0247 | 0.0000 | 0.0000 | 0.0032 | 0.0000 | 0.0000 | 0.0056 |
| **GSM2329058** | 0.2949 | 0.0000 | 0.0000 | 0.0123 | 0.0000 | 0.0000 | 0.0782 | 0.0623 | 0.0000 | 0.3583 | 0.0000 | 0.0130 | 0.0000 | 0.0852 | 0.0594 | 0.0277 | 0.0000 | 0.0000 | 0.0000 | 0.0017 | 0.0000 | 0.0069 |
| **GSM2329059** | 0.0242 | 0.0000 | 0.0000 | 0.0386 | 0.0000 | 0.0000 | 0.0000 | 0.1088 | 0.0000 | 0.2954 | 0.0000 | 0.1161 | 0.0000 | 0.2777 | 0.0856 | 0.0172 | 0.0000 | 0.0000 | 0.0000 | 0.0317 | 0.0000 | 0.0046 |
| **GSM2329060** | 0.0274 | 0.0000 | 0.0000 | 0.0234 | 0.0000 | 0.0004 | 0.0886 | 0.0471 | 0.0000 | 0.2969 | 0.0000 | 0.0000 | 0.0000 | 0.2699 | 0.1139 | 0.0991 | 0.0000 | 0.0000 | 0.0242 | 0.0000 | 0.0000 | 0.0090 |
| **GSM2329061** | 0.0053 | 0.0717 | 0.0000 | 0.0000 | 0.0009 | 0.0446 | 0.1298 | 0.0509 | 0.0000 | 0.1642 | 0.0000 | 0.0000 | 0.0000 | 0.3038 | 0.1372 | 0.0522 | 0.0004 | 0.0000 | 0.0323 | 0.0000 | 0.0000 | 0.0068 |
| **GSM2329062** | 0.3556 | 0.0000 | 0.0000 | 0.0000 | 0.0020 | 0.0000 | 0.0230 | 0.1854 | 0.0000 | 0.1611 | 0.0000 | 0.0284 | 0.0000 | 0.1338 | 0.0721 | 0.0308 | 0.0000 | 0.0000 | 0.0000 | 0.0000 | 0.0000 | 0.0078 |
| **GSM2329063** | 0.1541 | 0.0000 | 0.0000 | 0.0000 | 0.0196 | 0.1399 | 0.0654 | 0.0143 | 0.0000 | 0.1048 | 0.0054 | 0.0000 | 0.0000 | 0.3310 | 0.0678 | 0.0765 | 0.0080 | 0.0000 | 0.0131 | 0.0000 | 0.0000 | 0.0000 |
| **GSM2329064** | 0.0365 | 0.0000 | 0.0000 | 0.0614 | 0.0000 | 0.0165 | 0.0078 | 0.1269 | 0.0000 | 0.3428 | 0.0000 | 0.0028 | 0.0000 | 0.2117 | 0.1113 | 0.0618 | 0.0000 | 0.0000 | 0.0000 | 0.0204 | 0.0000 | 0.0000 |
| **GSM2329065** | 0.0315 | 0.0189 | 0.0000 | 0.0000 | 0.0739 | 0.0809 | 0.1828 | 0.0960 | 0.0000 | 0.2702 | 0.0000 | 0.0000 | 0.0000 | 0.0279 | 0.1146 | 0.0549 | 0.0000 | 0.0016 | 0.0000 | 0.0409 | 0.0000 | 0.0060 |
| **GSM2329066** | 0.0786 | 0.0000 | 0.0000 | 0.1079 | 0.0000 | 0.0000 | 0.0720 | 0.0312 | 0.0000 | 0.3023 | 0.0000 | 0.0000 | 0.0000 | 0.2420 | 0.1021 | 0.0473 | 0.0000 | 0.0000 | 0.0000 | 0.0134 | 0.0000 | 0.0033 |
| **GSM2329067** | 0.1587 | 0.0000 | 0.0000 | 0.0000 | 0.0075 | 0.0000 | 0.1211 | 0.0847 | 0.0000 | 0.1769 | 0.0000 | 0.0161 | 0.0000 | 0.0672 | 0.1728 | 0.0868 | 0.0157 | 0.0000 | 0.0000 | 0.0193 | 0.0612 | 0.0120 |
| **GSM2329068** | 0.1118 | 0.0000 | 0.0538 | 0.0702 | 0.0000 | 0.0000 | 0.0497 | 0.1237 | 0.0000 | 0.0903 | 0.0000 | 0.0097 | 0.0000 | 0.2311 | 0.1776 | 0.0630 | 0.0000 | 0.0000 | 0.0080 | 0.0000 | 0.0000 | 0.0109 |
| **GSM2329069** | 0.0000 | 0.0000 | 0.0281 | 0.1085 | 0.0000 | 0.0000 | 0.1752 | 0.0541 | 0.0000 | 0.3222 | 0.0000 | 0.0538 | 0.0000 | 0.0971 | 0.0772 | 0.0584 | 0.0000 | 0.0000 | 0.0000 | 0.0035 | 0.0051 | 0.0169 |
| **GSM2329070** | 0.3560 | 0.0000 | 0.0000 | 0.0262 | 0.0000 | 0.0000 | 0.0000 | 0.1040 | 0.0000 | 0.0545 | 0.0000 | 0.0252 | 0.0000 | 0.3361 | 0.0640 | 0.0279 | 0.0000 | 0.0000 | 0.0061 | 0.0000 | 0.0000 | 0.0000 |
| **GSM2329071** | 0.1202 | 0.0000 | 0.0090 | 0.1580 | 0.0000 | 0.0000 | 0.2105 | 0.0353 | 0.0000 | 0.2400 | 0.0000 | 0.0087 | 0.0000 | 0.0208 | 0.1361 | 0.0457 | 0.0014 | 0.0000 | 0.0059 | 0.0000 | 0.0085 | 0.0000 |
| **GSM2329072** | 0.2145 | 0.0000 | 0.0000 | 0.0518 | 0.0000 | 0.0015 | 0.1151 | 0.0943 | 0.0000 | 0.3091 | 0.0000 | 0.0152 | 0.0000 | 0.0529 | 0.0703 | 0.0501 | 0.0062 | 0.0000 | 0.0000 | 0.0000 | 0.0122 | 0.0068 |
| **GSM2329074** | 0.0234 | 0.0076 | 0.0000 | 0.0000 | 0.0000 | 0.0614 | 0.0158 | 0.1148 | 0.0000 | 0.3365 | 0.0000 | 0.0193 | 0.0000 | 0.2510 | 0.1172 | 0.0387 | 0.0000 | 0.0000 | 0.0000 | 0.0000 | 0.0069 | 0.0074 |
| **GSM2329075** | 0.0707 | 0.0000 | 0.0000 | 0.0000 | 0.0000 | 0.0533 | 0.0788 | 0.2899 | 0.0000 | 0.1411 | 0.0000 | 0.0009 | 0.0000 | 0.1182 | 0.0425 | 0.1834 | 0.0000 | 0.0000 | 0.0091 | 0.0000 | 0.0085 | 0.0036 |
| **GSM2329076** | 0.3565 | 0.0000 | 0.0000 | 0.0058 | 0.0000 | 0.0000 | 0.0494 | 0.2252 | 0.0000 | 0.1298 | 0.0000 | 0.0000 | 0.0000 | 0.1501 | 0.0400 | 0.0341 | 0.0000 | 0.0000 | 0.0091 | 0.0000 | 0.0000 | 0.0000 |
| **GSM2329077** | 0.2024 | 0.0000 | 0.0219 | 0.0000 | 0.0000 | 0.0961 | 0.0318 | 0.0410 | 0.0000 | 0.1649 | 0.0000 | 0.0026 | 0.0000 | 0.2290 | 0.0811 | 0.1064 | 0.0000 | 0.0000 | 0.0004 | 0.0024 | 0.0000 | 0.0201 |
| **GSM2329078** | 0.1431 | 0.0000 | 0.0000 | 0.1108 | 0.0000 | 0.0000 | 0.1176 | 0.0805 | 0.0000 | 0.2402 | 0.0000 | 0.0310 | 0.0000 | 0.0771 | 0.1258 | 0.0359 | 0.0149 | 0.0000 | 0.0000 | 0.0147 | 0.0000 | 0.0083 |
| **GSM2329079** | 0.3057 | 0.0000 | 0.0000 | 0.0000 | 0.0000 | 0.0561 | 0.0224 | 0.0517 | 0.0000 | 0.1188 | 0.0225 | 0.0000 | 0.0000 | 0.2747 | 0.0761 | 0.0635 | 0.0000 | 0.0000 | 0.0084 | 0.0000 | 0.0000 | 0.0000 |
| **GSM2329080** | 0.1202 | 0.0000 | 0.0000 | 0.0000 | 0.0000 | 0.1815 | 0.1446 | 0.0331 | 0.0000 | 0.0935 | 0.0000 | 0.0000 | 0.0000 | 0.3051 | 0.0251 | 0.0741 | 0.0033 | 0.0000 | 0.0095 | 0.0000 | 0.0036 | 0.0064 |
| **GSM2329082** | 0.0704 | 0.0000 | 0.0180 | 0.1078 | 0.0000 | 0.0000 | 0.1059 | 0.0556 | 0.0000 | 0.3110 | 0.0000 | 0.0054 | 0.0000 | 0.2525 | 0.0440 | 0.0263 | 0.0000 | 0.0000 | 0.0011 | 0.0000 | 0.0000 | 0.0020 |
| **GSM2329083** | 0.0000 | 0.0130 | 0.0017 | 0.1548 | 0.0000 | 0.0000 | 0.1813 | 0.0398 | 0.0000 | 0.2229 | 0.0000 | 0.0309 | 0.0000 | 0.1246 | 0.1789 | 0.0457 | 0.0000 | 0.0000 | 0.0000 | 0.0050 | 0.0000 | 0.0016 |
| **GSM2329084** | 0.0352 | 0.0000 | 0.0000 | 0.1087 | 0.0000 | 0.0000 | 0.1662 | 0.0491 | 0.0000 | 0.2109 | 0.0000 | 0.0073 | 0.0000 | 0.3142 | 0.0798 | 0.0234 | 0.0020 | 0.0000 | 0.0009 | 0.0000 | 0.0000 | 0.0024 |
| **GSM2329086** | 0.1656 | 0.0000 | 0.0000 | 0.0451 | 0.0000 | 0.0000 | 0.0829 | 0.0761 | 0.0000 | 0.2716 | 0.0000 | 0.0000 | 0.0000 | 0.2617 | 0.0528 | 0.0139 | 0.0000 | 0.0000 | 0.0303 | 0.0000 | 0.0000 | 0.0000 |
| **GSM2329087** | 0.0454 | 0.0000 | 0.0000 | 0.0778 | 0.0000 | 0.0000 | 0.0872 | 0.0598 | 0.0000 | 0.1246 | 0.0000 | 0.0021 | 0.0000 | 0.4275 | 0.1198 | 0.0348 | 0.0000 | 0.0000 | 0.0210 | 0.0000 | 0.0000 | 0.0000 |
| **GSM2329088** | 0.1342 | 0.0000 | 0.0000 | 0.1339 | 0.0000 | 0.0000 | 0.1400 | 0.0278 | 0.0000 | 0.3369 | 0.0000 | 0.0026 | 0.0000 | 0.1501 | 0.0108 | 0.0571 | 0.0000 | 0.0000 | 0.0000 | 0.0061 | 0.0000 | 0.0006 |
| **GSM2329089** | 0.0000 | 0.0702 | 0.0000 | 0.0114 | 0.0040 | 0.0000 | 0.0645 | 0.0498 | 0.0000 | 0.2365 | 0.0000 | 0.0000 | 0.0000 | 0.3871 | 0.1378 | 0.0187 | 0.0022 | 0.0000 | 0.0178 | 0.0000 | 0.0000 | 0.0000 |
| **GSM2329090** | 0.0032 | 0.0348 | 0.0154 | 0.0465 | 0.0000 | 0.0000 | 0.1952 | 0.1011 | 0.0000 | 0.2963 | 0.0000 | 0.0000 | 0.0000 | 0.1518 | 0.1270 | 0.0134 | 0.0000 | 0.0000 | 0.0000 | 0.0133 | 0.0000 | 0.0020 |
| **GSM2329091** | 0.0996 | 0.0000 | 0.0000 | 0.0443 | 0.0000 | 0.0000 | 0.1808 | 0.0623 | 0.0000 | 0.3547 | 0.0000 | 0.0000 | 0.0000 | 0.1412 | 0.0659 | 0.0419 | 0.0000 | 0.0000 | 0.0070 | 0.0000 | 0.0000 | 0.0022 |
| **GSM2329092** | 0.0403 | 0.0366 | 0.0233 | 0.0000 | 0.0518 | 0.0602 | 0.2768 | 0.0223 | 0.0000 | 0.1707 | 0.0151 | 0.0000 | 0.0000 | 0.1205 | 0.0719 | 0.0608 | 0.0218 | 0.0000 | 0.0000 | 0.0000 | 0.0135 | 0.0143 |
| **GSM2329093** | 0.2956 | 0.0000 | 0.0000 | 0.1251 | 0.0000 | 0.0000 | 0.0100 | 0.0675 | 0.0049 | 0.1225 | 0.0000 | 0.0670 | 0.0000 | 0.2373 | 0.0322 | 0.0060 | 0.0000 | 0.0000 | 0.0000 | 0.0319 | 0.0000 | 0.0000 |
| **GSM2329094** | 0.2046 | 0.0000 | 0.0000 | 0.0979 | 0.0000 | 0.0000 | 0.1205 | 0.0773 | 0.0000 | 0.2982 | 0.0000 | 0.0403 | 0.0000 | 0.0255 | 0.0700 | 0.0623 | 0.0029 | 0.0000 | 0.0000 | 0.0000 | 0.0006 | 0.0000 |
| **GSM2329095** | 0.3388 | 0.0000 | 0.0000 | 0.0719 | 0.0046 | 0.0000 | 0.0765 | 0.0314 | 0.0000 | 0.1956 | 0.0000 | 0.0000 | 0.0000 | 0.0842 | 0.1212 | 0.0597 | 0.0073 | 0.0000 | 0.0000 | 0.0051 | 0.0001 | 0.0036 |
| **GSM2329096** | 0.1582 | 0.0000 | 0.0000 | 0.0000 | 0.0945 | 0.0016 | 0.1311 | 0.1238 | 0.0000 | 0.2259 | 0.0000 | 0.0000 | 0.0000 | 0.1846 | 0.0324 | 0.0262 | 0.0000 | 0.0034 | 0.0107 | 0.0000 | 0.0000 | 0.0076 |
| **GSM2329097** | 0.0703 | 0.0000 | 0.0000 | 0.0000 | 0.0226 | 0.0000 | 0.0681 | 0.1045 | 0.0778 | 0.1514 | 0.0000 | 0.0000 | 0.0000 | 0.2915 | 0.1101 | 0.0567 | 0.0000 | 0.0000 | 0.0050 | 0.0046 | 0.0374 | 0.0000 |
| **GSM2329098** | 0.1263 | 0.0000 | 0.0000 | 0.1228 | 0.0000 | 0.0000 | 0.0670 | 0.0766 | 0.0000 | 0.2344 | 0.0000 | 0.0070 | 0.0000 | 0.2764 | 0.0519 | 0.0111 | 0.0000 | 0.0000 | 0.0000 | 0.0237 | 0.0000 | 0.0028 |
| **GSM2329099** | 0.3389 | 0.0000 | 0.0000 | 0.0576 | 0.0000 | 0.0000 | 0.0179 | 0.1266 | 0.0000 | 0.2165 | 0.0000 | 0.0413 | 0.0000 | 0.1026 | 0.0413 | 0.0414 | 0.0000 | 0.0000 | 0.0000 | 0.0159 | 0.0000 | 0.0000 |
| **GSM2329100** | 0.2146 | 0.0000 | 0.0000 | 0.0000 | 0.0000 | 0.0257 | 0.1585 | 0.0415 | 0.0000 | 0.2465 | 0.0000 | 0.0060 | 0.0000 | 0.1315 | 0.1192 | 0.0402 | 0.0000 | 0.0000 | 0.0000 | 0.0139 | 0.0000 | 0.0024 |
| **GSM2329101** | 0.0575 | 0.0000 | 0.0000 | 0.0000 | 0.0000 | 0.0160 | 0.0704 | 0.1242 | 0.0000 | 0.2473 | 0.0000 | 0.0117 | 0.0000 | 0.2064 | 0.0705 | 0.0000 | 0.0000 | 0.1577 | 0.0289 | 0.0000 | 0.0000 | 0.0093 |
| **GSM2329102** | 0.0213 | 0.0000 | 0.0000 | 0.1211 | 0.0000 | 0.0000 | 0.2193 | 0.0648 | 0.0000 | 0.2922 | 0.0000 | 0.0000 | 0.0000 | 0.1045 | 0.1137 | 0.0466 | 0.0053 | 0.0000 | 0.0000 | 0.0043 | 0.0000 | 0.0069 |
| **GSM2329103** | 0.0728 | 0.0000 | 0.0314 | 0.0849 | 0.0000 | 0.0000 | 0.1233 | 0.0805 | 0.0000 | 0.1829 | 0.0000 | 0.0104 | 0.0000 | 0.2310 | 0.1101 | 0.0399 | 0.0096 | 0.0000 | 0.0000 | 0.0198 | 0.0000 | 0.0034 |
| **GSM2329104** | 0.0504 | 0.0030 | 0.0000 | 0.0000 | 0.0000 | 0.1021 | 0.1534 | 0.0512 | 0.0000 | 0.2880 | 0.0000 | 0.0000 | 0.0000 | 0.1939 | 0.1228 | 0.0292 | 0.0000 | 0.0000 | 0.0060 | 0.0000 | 0.0000 | 0.0000 |
| **GSM2329105** | 0.0976 | 0.0229 | 0.0000 | 0.0000 | 0.0000 | 0.0000 | 0.0927 | 0.0904 | 0.0000 | 0.1813 | 0.0000 | 0.0345 | 0.0000 | 0.3344 | 0.1185 | 0.0188 | 0.0000 | 0.0000 | 0.0041 | 0.0000 | 0.0000 | 0.0048 |
| **GSM2329106** | 0.0415 | 0.0000 | 0.0000 | 0.0331 | 0.0000 | 0.0000 | 0.0440 | 0.0800 | 0.0000 | 0.1198 | 0.0000 | 0.0340 | 0.0000 | 0.5838 | 0.0450 | 0.0101 | 0.0018 | 0.0000 | 0.0033 | 0.0000 | 0.0037 | 0.0000 |
| **GSM2329107** | 0.0658 | 0.0038 | 0.0000 | 0.0081 | 0.0000 | 0.0240 | 0.0879 | 0.1383 | 0.0000 | 0.1632 | 0.0000 | 0.0000 | 0.0000 | 0.3025 | 0.1325 | 0.0406 | 0.0202 | 0.0000 | 0.0001 | 0.0000 | 0.0000 | 0.0129 |
| **GSM2329108** | 0.0241 | 0.0339 | 0.0000 | 0.0504 | 0.0000 | 0.0000 | 0.0522 | 0.1013 | 0.0000 | 0.4118 | 0.0000 | 0.0000 | 0.0000 | 0.1497 | 0.1215 | 0.0326 | 0.0000 | 0.0000 | 0.0000 | 0.0143 | 0.0000 | 0.0084 |
| **GSM2329109** | 0.0368 | 0.0000 | 0.0016 | 0.1231 | 0.0000 | 0.0000 | 0.0728 | 0.0709 | 0.0000 | 0.2182 | 0.0000 | 0.0303 | 0.0000 | 0.3035 | 0.1161 | 0.0237 | 0.0031 | 0.0000 | 0.0000 | 0.0000 | 0.0000 | 0.0000 |
| **GSM2329110** | 0.1182 | 0.0000 | 0.0000 | 0.0000 | 0.0023 | 0.0726 | 0.2455 | 0.0458 | 0.0000 | 0.2906 | 0.0000 | 0.0000 | 0.0000 | 0.0691 | 0.1192 | 0.0291 | 0.0000 | 0.0000 | 0.0000 | 0.0000 | 0.0000 | 0.0076 |
| **GSM2329111** | 0.2530 | 0.0000 | 0.0000 | 0.0000 | 0.0000 | 0.0506 | 0.0274 | 0.2195 | 0.0000 | 0.2285 | 0.0000 | 0.0000 | 0.0000 | 0.1528 | 0.0172 | 0.0316 | 0.0000 | 0.0000 | 0.0170 | 0.0000 | 0.0000 | 0.0024 |
| **GSM2329112** | 0.1363 | 0.0000 | 0.2593 | 0.0000 | 0.0000 | 0.0000 | 0.0000 | 0.1228 | 0.0000 | 0.1353 | 0.0000 | 0.0000 | 0.0000 | 0.1621 | 0.1014 | 0.0687 | 0.0000 | 0.0000 | 0.0000 | 0.0141 | 0.0000 | 0.0000 |
| **GSM2329113** | 0.0448 | 0.0590 | 0.0000 | 0.0000 | 0.0000 | 0.0000 | 0.1779 | 0.0627 | 0.0000 | 0.1583 | 0.0000 | 0.0109 | 0.0000 | 0.3231 | 0.0131 | 0.0251 | 0.0000 | 0.0752 | 0.0000 | 0.0159 | 0.0032 | 0.0307 |
| **GSM2329114** | 0.0092 | 0.0101 | 0.0000 | 0.0000 | 0.0038 | 0.0000 | 0.2336 | 0.1254 | 0.0000 | 0.2039 | 0.0000 | 0.0000 | 0.0000 | 0.2449 | 0.0826 | 0.0254 | 0.0000 | 0.0535 | 0.0039 | 0.0000 | 0.0000 | 0.0036 |
| **GSM2329115** | 0.2221 | 0.1220 | 0.0000 | 0.0000 | 0.0925 | 0.0000 | 0.0175 | 0.1097 | 0.0000 | 0.1677 | 0.0000 | 0.0000 | 0.0000 | 0.2080 | 0.0285 | 0.0262 | 0.0000 | 0.0000 | 0.0049 | 0.0000 | 0.0009 | 0.0000 |
| **GSM2329116** | 0.1325 | 0.0000 | 0.0000 | 0.0000 | 0.0000 | 0.0000 | 0.0238 | 0.0944 | 0.0000 | 0.1332 | 0.0000 | 0.0000 | 0.0000 | 0.4553 | 0.0909 | 0.0000 | 0.0000 | 0.0678 | 0.0000 | 0.0019 | 0.0000 | 0.0000 |
| **GSM2329117** | 0.0202 | 0.0000 | 0.0000 | 0.0382 | 0.0000 | 0.0000 | 0.1508 | 0.0962 | 0.0000 | 0.2012 | 0.0000 | 0.0000 | 0.0000 | 0.3845 | 0.0864 | 0.0000 | 0.0000 | 0.0149 | 0.0076 | 0.0000 | 0.0000 | 0.0000 |
| **GSM2329118** | 0.0099 | 0.0000 | 0.0058 | 0.0945 | 0.0000 | 0.0000 | 0.0994 | 0.0727 | 0.0000 | 0.2818 | 0.0000 | 0.0084 | 0.0000 | 0.2833 | 0.1177 | 0.0161 | 0.0064 | 0.0000 | 0.0000 | 0.0000 | 0.0000 | 0.0040 |
| **GSM2329119** | 0.0987 | 0.0000 | 0.0100 | 0.1926 | 0.0000 | 0.0000 | 0.1314 | 0.0357 | 0.0000 | 0.2320 | 0.0000 | 0.0249 | 0.0000 | 0.0132 | 0.1797 | 0.0492 | 0.0125 | 0.0000 | 0.0007 | 0.0000 | 0.0123 | 0.0070 |
| **GSM2329120** | 0.2222 | 0.0000 | 0.0000 | 0.0523 | 0.0000 | 0.0000 | 0.0891 | 0.1174 | 0.0000 | 0.2080 | 0.0000 | 0.0189 | 0.0000 | 0.1471 | 0.0620 | 0.0660 | 0.0101 | 0.0000 | 0.0000 | 0.0071 | 0.0000 | 0.0000 |
| **GSM2329121** | 0.0152 | 0.0000 | 0.0000 | 0.2751 | 0.0000 | 0.0000 | 0.1081 | 0.0776 | 0.0000 | 0.2807 | 0.0000 | 0.0000 | 0.0000 | 0.0756 | 0.0789 | 0.0339 | 0.0000 | 0.0000 | 0.0000 | 0.0345 | 0.0004 | 0.0199 |
| **GSM2329122** | 0.1337 | 0.0000 | 0.0845 | 0.0238 | 0.0000 | 0.0000 | 0.0297 | 0.0861 | 0.0000 | 0.0905 | 0.0000 | 0.0413 | 0.0000 | 0.4091 | 0.0962 | 0.0047 | 0.0000 | 0.0000 | 0.0004 | 0.0000 | 0.0000 | 0.0000 |
| **GSM2329123** | 0.2339 | 0.0000 | 0.0000 | 0.0000 | 0.0264 | 0.0902 | 0.0776 | 0.1068 | 0.0000 | 0.3168 | 0.0000 | 0.0018 | 0.0000 | 0.0458 | 0.0571 | 0.0131 | 0.0034 | 0.0000 | 0.0000 | 0.0206 | 0.0000 | 0.0066 |
| **GSM2329124** | 0.1371 | 0.0559 | 0.0000 | 0.0000 | 0.0000 | 0.0287 | 0.0579 | 0.1150 | 0.0000 | 0.3690 | 0.0000 | 0.0362 | 0.0000 | 0.0651 | 0.0864 | 0.0420 | 0.0000 | 0.0000 | 0.0000 | 0.0043 | 0.0013 | 0.0011 |
| **GSM2329125** | 0.3578 | 0.0000 | 0.0000 | 0.0975 | 0.0000 | 0.0000 | 0.0196 | 0.1109 | 0.0000 | 0.0952 | 0.0000 | 0.0276 | 0.0000 | 0.1258 | 0.0340 | 0.0732 | 0.0000 | 0.0000 | 0.0000 | 0.0584 | 0.0000 | 0.0000 |
| **GSM2329126** | 0.1303 | 0.0000 | 0.0000 | 0.1021 | 0.0000 | 0.0000 | 0.0468 | 0.1206 | 0.0000 | 0.2133 | 0.0000 | 0.0011 | 0.0000 | 0.1959 | 0.1830 | 0.0061 | 0.0000 | 0.0000 | 0.0000 | 0.0000 | 0.0000 | 0.0008 |
| **GSM2329127** | 0.2775 | 0.0000 | 0.0000 | 0.0000 | 0.0000 | 0.0000 | 0.2578 | 0.0538 | 0.0000 | 0.1806 | 0.0000 | 0.0066 | 0.0000 | 0.0428 | 0.0574 | 0.0592 | 0.0078 | 0.0000 | 0.0359 | 0.0000 | 0.0000 | 0.0204 |
| **GSM2329128** | 0.0187 | 0.0886 | 0.0000 | 0.0000 | 0.0000 | 0.0000 | 0.1379 | 0.0698 | 0.0000 | 0.1314 | 0.0000 | 0.0000 | 0.0000 | 0.3987 | 0.1328 | 0.0201 | 0.0019 | 0.0000 | 0.0000 | 0.0000 | 0.0000 | 0.0002 |
| **GSM2329129** | 0.0018 | 0.0357 | 0.0557 | 0.2393 | 0.0000 | 0.0000 | 0.2757 | 0.0339 | 0.0000 | 0.1393 | 0.0000 | 0.0234 | 0.0000 | 0.0195 | 0.0967 | 0.0491 | 0.0108 | 0.0000 | 0.0052 | 0.0000 | 0.0086 | 0.0052 |
| **GSM2329130** | 0.5151 | 0.0000 | 0.0000 | 0.0000 | 0.0000 | 0.0000 | 0.0450 | 0.0676 | 0.0000 | 0.1320 | 0.0000 | 0.0026 | 0.0000 | 0.1228 | 0.0507 | 0.0617 | 0.0000 | 0.0000 | 0.0009 | 0.0000 | 0.0000 | 0.0015 |
| **GSM2329131** | 0.2712 | 0.0000 | 0.0000 | 0.0000 | 0.0527 | 0.0212 | 0.0904 | 0.0875 | 0.0000 | 0.2671 | 0.0000 | 0.0000 | 0.0000 | 0.1463 | 0.0190 | 0.0229 | 0.0000 | 0.0000 | 0.0000 | 0.0146 | 0.0000 | 0.0071 |
| **GSM2329132** | 0.1153 | 0.0000 | 0.0000 | 0.0000 | 0.0000 | 0.0110 | 0.0953 | 0.1138 | 0.0000 | 0.2503 | 0.0000 | 0.0051 | 0.0000 | 0.3331 | 0.0725 | 0.0037 | 0.0000 | 0.0000 | 0.0000 | 0.0000 | 0.0000 | 0.0001 |
| **GSM2329133** | 0.1895 | 0.0611 | 0.0000 | 0.0000 | 0.0000 | 0.1482 | 0.0000 | 0.1825 | 0.0000 | 0.1110 | 0.0000 | 0.0000 | 0.0000 | 0.1746 | 0.0714 | 0.0335 | 0.0069 | 0.0000 | 0.0000 | 0.0172 | 0.0040 | 0.0000 |
| **GSM2329134** | 0.1095 | 0.0000 | 0.0000 | 0.0000 | 0.0000 | 0.0000 | 0.0752 | 0.1082 | 0.0000 | 0.2240 | 0.0000 | 0.0379 | 0.0000 | 0.3053 | 0.1041 | 0.0212 | 0.0000 | 0.0000 | 0.0000 | 0.0128 | 0.0000 | 0.0018 |
| **GSM2329135** | 0.4212 | 0.0000 | 0.0000 | 0.0000 | 0.0000 | 0.0321 | 0.0255 | 0.0777 | 0.0000 | 0.1489 | 0.0000 | 0.0004 | 0.0000 | 0.1686 | 0.0538 | 0.0718 | 0.0000 | 0.0000 | 0.0000 | 0.0000 | 0.0000 | 0.0000 |
| **GSM2329136** | 0.1153 | 0.0000 | 0.0213 | 0.0000 | 0.0000 | 0.0818 | 0.0654 | 0.0737 | 0.0000 | 0.3720 | 0.0000 | 0.0141 | 0.0000 | 0.0679 | 0.0779 | 0.0614 | 0.0000 | 0.0000 | 0.0000 | 0.0386 | 0.0000 | 0.0107 |
| **GSM2329137** | 0.0336 | 0.0891 | 0.0175 | 0.0000 | 0.0000 | 0.0578 | 0.0462 | 0.1052 | 0.0000 | 0.2676 | 0.0000 | 0.0000 | 0.0000 | 0.1934 | 0.0711 | 0.0419 | 0.0000 | 0.0000 | 0.0000 | 0.0676 | 0.0003 | 0.0086 |
| **GSM2329138** | 0.2063 | 0.0000 | 0.0000 | 0.0000 | 0.0000 | 0.0000 | 0.1213 | 0.0722 | 0.0000 | 0.1975 | 0.0000 | 0.0219 | 0.0000 | 0.2729 | 0.0700 | 0.0295 | 0.0000 | 0.0000 | 0.0064 | 0.0000 | 0.0000 | 0.0021 |
| **GSM2329139** | 0.3948 | 0.0000 | 0.0000 | 0.0583 | 0.0027 | 0.0000 | 0.0155 | 0.0326 | 0.0015 | 0.1868 | 0.0000 | 0.0000 | 0.0000 | 0.1668 | 0.0396 | 0.0948 | 0.0000 | 0.0000 | 0.0000 | 0.0007 | 0.0029 | 0.0030 |

**Supplementary Material Table S2: 14 candidate hub genes by setting Gene-Significance and Module-Membership.**

| **Gene** | **MMmagenta** | **MMpink** | **MMyellow** | **MMred** | **MMtan** | **MMcyan** | **MMblack** | **MMsalmon** | **MMblue** | **MMpurple** | **MMbrown** | **MMgreenyellow** | **MMgrey** | **GS.T cells CD8** | **GS.T cells CD4 naive** | **GS.T cells CD4 memory resting** | **GS.T cells CD4 memory activated** | **GS.T cells follicular helper** | **GS.T cells regulatory (Tregs)** | **GS.T cells gamma delta** |
| --- | --- | --- | --- | --- | --- | --- | --- | --- | --- | --- | --- | --- | --- | --- | --- | --- | --- | --- | --- | --- |
| **C2** | -0.132147287 | -0.18744 | -0.1701304 | -0.22236 | 0.065661 | -0.11112 | 0.3227719 | -0.17588468 | 0.236607 | 0.08994572 | 0.88186234 | 0.50058057 | -0.09227 | 0.343656345 | -0.163532885 | -0.294940936 | 0.496607973 | -0.262261497 | -0.10042414 | 0.246163413 |
| **FCER1G** | -0.102109664 | -0.14865 | -0.1878577 | -0.41719 | -0.10431 | -0.07255 | 0.2810246 | -0.11123189 | 0.331048 | 0.26110844 | 0.88159952 | 0.52749081 | -0.08132 | 0.332350091 | -0.111866316 | -0.187749668 | 0.465499987 | -0.299106954 | -0.144059649 | 0.336840523 |
| **DUSP3** | 0.083815015 | -0.01552 | -0.0720407 | -0.51994 | -0.14091 | -0.06555 | 0.1541013 | -0.18206429 | 0.28764 | 0.21684793 | 0.81144551 | 0.277302789 | -0.0776 | 0.375787381 | -0.085386257 | -0.125314379 | 0.584388681 | -0.364434402 | -0.1226168 | 0.228607405 |
| **GBP1** | -0.09446534 | -0.06178 | -0.3249936 | -0.21426 | -0.0641 | -0.0945 | 0.4461571 | -0.04878849 | 0.270854 | 0.24903379 | 0.85430436 | 0.459538049 | -0.00099 | 0.331333272 | -0.046004499 | -0.215584081 | 0.475380441 | -0.238394113 | -0.130539011 | 0.420224368 |
| **SERPINA1** | -0.195726157 | -0.22174 | -0.1683804 | -0.33174 | 0.047471 | -0.02914 | 0.2564911 | -0.14169106 | 0.364479 | 0.19156756 | 0.85854741 | 0.623217749 | -0.07706 | 0.226994691 | -0.129268916 | -0.234139095 | 0.424435366 | -0.242199015 | -0.107961751 | 0.242294862 |
| **SLC31A2** | -0.087545314 | -0.11404 | -0.3337245 | -0.37987 | -0.10331 | -0.03849 | 0.386015 | -0.03699974 | 0.394993 | 0.35649969 | 0.90608537 | 0.680797083 | -0.07419 | 0.290972194 | -0.108662276 | -0.180741045 | 0.412763411 | -0.222096917 | -0.161330933 | 0.345725489 |
| **VAMP5** | -0.063379996 | -0.15084 | -0.2897737 | -0.38737 | -0.09846 | -0.04403 | 0.4781984 | 0.04433263 | 0.163483 | 0.25704259 | 0.86959044 | 0.35341465 | -0.15005 | 0.349077987 | -0.028138696 | -0.165659744 | 0.537135884 | -0.223551122 | -0.181636552 | 0.432298447 |
| **CD33** | -0.051788388 | -0.10683 | -0.2050881 | -0.33039 | -0.07009 | -0.15306 | 0.2986735 | -0.04169205 | 0.257385 | 0.23447004 | 0.82079157 | 0.495948736 | -0.20294 | 0.287288859 | -0.121200327 | -0.155847828 | 0.40416075 | -0.278018583 | -0.145164426 | 0.277722513 |
| **LILRB1** | -0.178365036 | -0.21999 | -0.1652377 | -0.21422 | 0.136355 | -0.0474 | 0.2212262 | -0.2083314 | 0.373918 | 0.1261942 | 0.82932991 | 0.662155374 | 0.001497 | 0.243088855 | -0.172005138 | -0.269918712 | 0.406391249 | -0.241077927 | -0.127496786 | 0.13781954 |
| **EMR2** | -0.110659057 | -0.17096 | -0.2339492 | -0.23033 | 0.058615 | -0.1102 | 0.3291915 | -0.02089988 | 0.342807 | 0.26588079 | 0.83300585 | 0.55725793 | -0.16553 | 0.120269167 | -0.038814828 | -0.16204423 | 0.470823916 | -0.222807354 | -0.120495632 | 0.207314011 |
| **LILRB2** | -0.129157966 | -0.10101 | -0.0837286 | -0.30299 | 0.034723 | -0.06623 | 0.2209336 | -0.13993287 | 0.196254 | 0.12938321 | 0.84188509 | 0.283421439 | -0.15578 | 0.303371213 | -0.13020678 | -0.195911361 | 0.58210954 | -0.390190601 | -0.199009647 | 0.288344985 |
| **LAIR1** | -0.155368598 | -0.19027 | -0.2871816 | -0.29852 | -0.02787 | -0.09311 | 0.3996187 | 0.02722741 | 0.30097 | 0.28836859 | 0.82497705 | 0.520036854 | -0.10736 | 0.285077665 | -0.110801475 | -0.201386655 | 0.400175565 | -0.162675932 | -0.17832105 | 0.321996575 |
| **C3AR1** | -0.065840478 | -0.06183 | -0.2890754 | -0.40925 | -0.15433 | -0.04957 | 0.2643945 | -0.06110862 | 0.389709 | 0.37373809 | 0.84390639 | 0.474825162 | -0.08346 | 0.31330197 | -0.171855955 | -0.130491319 | 0.441507516 | -0.293139996 | -0.183237559 | 0.315864684 |
| **LILRB3** | -0.166237384 | -0.15659 | -0.0765867 | -0.25123 | 0.102605 | -0.06481 | 0.1671591 | -0.28446243 | 0.304028 | 0.04661335 | 0.85518541 | 0.472402252 | -0.02398 | 0.354336743 | -0.200358233 | -0.255843333 | 0.473303928 | -0.33753543 | -0.136651489 | 0.162329025 |

**Supplementary Material Table S3: The expression of FCER1G, distributions of the risk scores between high-risk and low-risk patients**

| **ID** | **The expression of FCER1G** | **RiskScore** | **RiskGroup** |
| --- | --- | --- | --- |
| GSM2329125 | 4.372808941 | 0.649674346 | low |
| GSM2328977 | 5.0639283 | 0.752354917 | low |
| GSM2328968 | 5.367962106 | 0.797525645 | low |
| GSM2329015 | 5.406633688 | 0.803271136 | low |
| GSM2328941 | 5.752952351 | 0.854724185 | low |
| GSM2329079 | 5.79999358 | 0.861713166 | low |
| GSM2329076 | 5.864533354 | 0.871301931 | low |
| GSM2329135 | 5.908836867 | 0.877884166 | low |
| GSM2329055 | 6.067588386 | 0.901470101 | low |
| GSM2329099 | 6.071775022 | 0.902092116 | low |
| GSM2329011 | 6.094131827 | 0.905413697 | low |
| GSM2328988 | 6.168236397 | 0.916423517 | low |
| GSM2328918 | 6.191512257 | 0.919881644 | low |
| GSM2328975 | 6.216588045 | 0.923607189 | low |
| GSM2328921 | 6.267474096 | 0.931167401 | low |
| GSM2328974 | 6.357422663 | 0.944531185 | low |
| GSM2329022 | 6.383805407 | 0.948450907 | low |
| GSM2329097 | 6.395265485 | 0.950153546 | low |
| GSM2329115 | 6.417862178 | 0.953510769 | low |
| GSM2329019 | 6.534210112 | 0.970796744 | low |
| GSM2329017 | 6.541132254 | 0.971825177 | low |
| GSM2328969 | 6.556052672 | 0.974041924 | low |
| GSM2329130 | 6.590487878 | 0.979158011 | low |
| GSM2329070 | 6.66442572 | 0.990143059 | low |
| GSM2328971 | 6.746377058 | 1.002318684 | low |
| GSM2328992 | 6.761195973 | 1.004520351 | low |
| GSM2329085 | 6.778312737 | 1.007063413 | low |
| GSM2328973 | 6.817517188 | 1.012888073 | low |
| GSM2328980 | 6.829411877 | 1.014655284 | low |
| GSM2329111 | 6.870594958 | 1.020773912 | low |
| GSM2328928 | 6.877846473 | 1.021851279 | low |
| GSM2329028 | 6.907372779 | 1.026238044 | low |
| GSM2328946 | 6.967200556 | 1.035126741 | low |
| GSM2329030 | 7.056799108 | 1.048438523 | low |
| GSM2329025 | 7.086455473 | 1.052844611 | low |
| GSM2328943 | 7.157821932 | 1.063447625 | low |
| GSM2329044 | 7.162194401 | 1.064097249 | low |
| GSM2329006 | 7.208439565 | 1.070967958 | low |
| GSM2328985 | 7.272850992 | 1.080537654 | low |
| GSM2329033 | 7.272850992 | 1.080537654 | low |
| GSM2329054 | 7.275759048 | 1.080969708 | low |
| GSM2328940 | 7.33642648 | 1.089983153 | low |
| GSM2328963 | 7.413930769 | 1.101498074 | low |
| GSM2329073 | 7.496297817 | 1.113735461 | low |
| GSM2328930 | 7.502249866 | 1.114619766 | low |
| GSM2329139 | 7.508394246 | 1.115532645 | low |
| GSM2328978 | 7.521256207 | 1.117443564 | low |
| GSM2328920 | 7.532245854 | 1.119076312 | low |
| GSM2329048 | 7.533745399 | 1.119299101 | low |
| GSM2329037 | 7.552527252 | 1.122089547 | low |
| GSM2328979 | 7.554012999 | 1.122310287 | low |
| GSM2329095 | 7.558588624 | 1.122990094 | low |
| GSM2328951 | 7.560071719 | 1.123210439 | low |
| GSM2329131 | 7.560071719 | 1.123210439 | low |
| GSM2329127 | 7.585249752 | 1.126951175 | low |
| GSM2328983 | 7.608201633 | 1.130361168 | low |
| GSM2329120 | 7.639552728 | 1.135019044 | low |
| GSM2329010 | 7.647542592 | 1.136206109 | low |
| GSM2329122 | 7.680122466 | 1.141046547 | low |
| GSM2329026 | 7.683337972 | 1.141524279 | low |
| GSM2329041 | 7.701560182 | 1.144231578 | low |
| GSM2328950 | 7.712536662 | 1.145862369 | low |
| GSM2328965 | 7.717419724 | 1.146587853 | low |
| GSM2329075 | 7.722364053 | 1.147322439 | low |
| GSM2328991 | 7.741385345 | 1.150148459 | low |
| GSM2329094 | 7.76210652 | 1.153227033 | low |
| GSM2329000 | 7.763694243 | 1.153462923 | low |
| GSM2329106 | 7.768170519 | 1.154127969 | low |
| GSM2329057 | 7.835203483 | 1.164087151 | low |
| GSM2329124 | 7.862781978 | 1.168184526 | low |
| GSM2329008 | 7.902365437 | 1.174065496 | low |
| GSM2329058 | 7.916542891 | 1.17617186 | low |
| GSM2328966 | 7.918177204 | 1.176414673 | low |
| GSM2329001 | 7.929317194 | 1.178069757 | low |
| GSM2328993 | 7.934163863 | 1.178789833 | low |
| GSM2329101 | 7.960434108 | 1.18269284 | low |
| GSM2329052 | 7.962181126 | 1.182952397 | low |
| GSM2328936 | 7.965252505 | 1.183408716 | low |
| GSM2329064 | 7.99370227 | 1.187635537 | low |
| GSM2329137 | 8.036126712 | 1.193938596 | low |
| GSM2328956 | 8.056566269 | 1.19697533 | low |
| GSM2329123 | 8.067654083 | 1.198622662 | low |
| GSM2328942 | 8.080150864 | 1.200479326 | low |
| GSM2329009 | 8.104027702 | 1.204026741 | low |
| GSM2328981 | 8.116681369 | 1.205906714 | low |
| GSM2329039 | 8.118188948 | 1.206130697 | low |
| GSM2328929 | 8.121599191 | 1.206637362 | low |
| GSM2328938 | 8.124984653 | 1.207140345 | low |
| GSM2328962 | 8.139751561 | 1.209334285 | low |
| GSM2328934 | 8.153936692 | 1.21144179 | low |
| GSM2328961 | 8.222272866 | 1.221594591 | low |
| GSM2329031 | 8.247872651 | 1.225397987 | low |
| GSM2329049 | 8.24932081 | 1.225613142 | low |
| GSM2329093 | 8.276891939 | 1.229709423 | low |
| GSM2328944 | 8.294519635 | 1.232328394 | low |
| GSM2328935 | 8.302662328 | 1.233538166 | low |
| GSM2328984 | 8.310750064 | 1.234739772 | low |
| GSM2329016 | 8.338556454 | 1.238871006 | low |
| GSM2329062 | 8.360735095 | 1.242166118 | low |
| GSM2329077 | 8.364066832 | 1.242661119 | low |
| GSM2329116 | 8.382770668 | 1.245439974 | low |
| GSM2329126 | 8.398866279 | 1.247831321 | low |
| GSM2329063 | 8.425842819 | 1.251839264 | low |
| GSM2329018 | 8.446347892 | 1.254885731 | low |
| GSM2329035 | 8.446347892 | 1.254885731 | low |
| GSM2329091 | 8.498629637 | 1.262653303 | low |
| GSM2329068 | 8.5023628 | 1.263207945 | low |
| GSM2329104 | 8.512491499 | 1.264712779 | low |
| GSM2329074 | 8.539855331 | 1.268778262 | low |
| GSM2329046 | 8.583225931 | 1.275221893 | low |
| GSM2328922 | 8.597098322 | 1.277282934 | high |
| GSM2329132 | 8.597098322 | 1.277282934 | high |
| GSM2329112 | 8.616141784 | 1.280112247 | high |
| GSM2329140 | 8.647871529 | 1.28482638 | high |
| GSM2328953 | 8.653600321 | 1.285677515 | high |
| GSM2329003 | 8.653600321 | 1.285677515 | high |
| GSM2328999 | 8.655246701 | 1.28592212 | high |
| GSM2329072 | 8.693945556 | 1.291671663 | high |
| GSM2329081 | 8.704397913 | 1.293224584 | high |
| GSM2329096 | 8.718161101 | 1.2952694 | high |
| GSM2329053 | 8.734694636 | 1.297725811 | high |
| GSM2329105 | 8.758664769 | 1.301287087 | high |
| GSM2329092 | 8.775311306 | 1.303760286 | high |
| GSM2329045 | 8.777204228 | 1.30404152 | high |
| GSM2329040 | 8.796046643 | 1.306840964 | high |
| GSM2329082 | 8.799805847 | 1.307399474 | high |
| GSM2329027 | 8.82636633 | 1.311345603 | high |
| GSM2329086 | 8.842178798 | 1.313694883 | high |
| GSM2328990 | 8.860121305 | 1.316360626 | high |
| GSM2328954 | 8.877262761 | 1.318907357 | high |
| GSM2329098 | 8.877262761 | 1.318907357 | high |
| GSM2328931 | 8.880937771 | 1.319453358 | high |
| GSM2328923 | 8.930489032 | 1.326815258 | high |
| GSM2329080 | 8.982620416 | 1.334560491 | high |
| GSM2328952 | 9.007117087 | 1.338199996 | high |
| GSM2329088 | 9.080081899 | 1.34904048 | high |
| GSM2328937 | 9.10460169 | 1.35268342 | high |
| GSM2329021 | 9.114489049 | 1.354152398 | high |
| GSM2329107 | 9.124125674 | 1.355584125 | high |
| GSM2329012 | 9.127701941 | 1.356115456 | high |
| GSM2328989 | 9.13380165 | 1.357021698 | high |
| GSM2328982 | 9.145638109 | 1.358780258 | high |
| GSM2328933 | 9.167303225 | 1.361999074 | high |
| GSM2329113 | 9.175043493 | 1.363149057 | high |
| GSM2329089 | 9.18308723 | 1.364344126 | high |
| GSM2329071 | 9.200950767 | 1.366998137 | high |
| GSM2328955 | 9.259340064 | 1.375673116 | high |
| GSM2328972 | 9.274312821 | 1.37789764 | high |
| GSM2329136 | 9.284053875 | 1.379344882 | high |
| GSM2328998 | 9.299858961 | 1.381693066 | high |
| GSM2329043 | 9.299858961 | 1.381693066 | high |
| GSM2328959 | 9.317818439 | 1.38436133 | high |
| GSM2329138 | 9.337286242 | 1.387253689 | high |
| GSM2329038 | 9.355161434 | 1.389909431 | high |
| GSM2329128 | 9.360755542 | 1.390740556 | high |
| GSM2329029 | 9.364542052 | 1.391303123 | high |
| GSM2328995 | 9.38036074 | 1.393653328 | high |
| GSM2329100 | 9.388885962 | 1.394919932 | high |
| GSM2328939 | 9.395034395 | 1.395833413 | high |
| GSM2329108 | 9.401514731 | 1.396796206 | high |
| GSM2329134 | 9.405796109 | 1.397432296 | high |
| GSM2328958 | 9.418000506 | 1.39924552 | high |
| GSM2329014 | 9.418000506 | 1.39924552 | high |
| GSM2329047 | 9.432374603 | 1.4013811 | high |
| GSM2329060 | 9.454407865 | 1.404654613 | high |
| GSM2329110 | 9.46620556 | 1.406407413 | high |
| GSM2328996 | 9.475020112 | 1.407717003 | high |
| GSM2329032 | 9.485007061 | 1.409200778 | high |
| GSM2329036 | 9.4873026 | 1.40954183 | high |
| GSM2329024 | 9.504198849 | 1.412052129 | high |
| GSM2329051 | 9.528827061 | 1.415711177 | high |
| GSM2328967 | 9.538850224 | 1.417200332 | high |
| GSM2328926 | 9.540745456 | 1.417481909 | high |
| GSM2328948 | 9.540745456 | 1.417481909 | high |
| GSM2329020 | 9.542777651 | 1.417783835 | high |
| GSM2329087 | 9.562459811 | 1.420708042 | high |
| GSM2328927 | 9.571173542 | 1.422002653 | high |
| GSM2329023 | 9.577448587 | 1.422934945 | high |
| GSM2328957 | 9.623902867 | 1.429836722 | high |
| GSM2328947 | 9.665604254 | 1.436032356 | high |
| GSM2329004 | 9.667935051 | 1.436378646 | high |
| GSM2328960 | 9.68790417 | 1.439345486 | high |
| GSM2329103 | 9.747758384 | 1.44823811 | high |
| GSM2329059 | 9.763783295 | 1.450618953 | high |
| GSM2329078 | 9.763783295 | 1.450618953 | high |
| GSM2328932 | 9.768190511 | 1.45127374 | high |
| GSM2329067 | 9.847126466 | 1.463001365 | high |
| GSM2328986 | 9.849545645 | 1.463360786 | high |
| GSM2328924 | 9.852094321 | 1.463739446 | high |
| GSM2329065 | 9.85459593 | 1.464111114 | high |
| GSM2328987 | 9.857251991 | 1.464505728 | high |
| GSM2329129 | 9.859948262 | 1.464906317 | high |
| GSM2328964 | 9.869949007 | 1.466392142 | high |
| GSM2329061 | 9.872623015 | 1.466789423 | high |
| GSM2329117 | 9.915814379 | 1.473206424 | high |
| GSM2329002 | 9.940789556 | 1.476917021 | high |
| GSM2329005 | 10.05827672 | 1.494372254 | high |
| GSM2329050 | 10.06096148 | 1.494771132 | high |
| GSM2329013 | 10.0634879 | 1.495146486 | high |
| GSM2329114 | 10.09912194 | 1.500440685 | high |
| GSM2329084 | 10.10224665 | 1.500904928 | high |
| GSM2329034 | 10.11253161 | 1.502432979 | high |
| GSM2329042 | 10.15133333 | 1.508197805 | high |
| GSM2329066 | 10.17993393 | 1.512447036 | high |
| GSM2329056 | 10.19957528 | 1.515365179 | high |
| GSM2328976 | 10.28721631 | 1.528386129 | high |
| GSM2328945 | 10.56193403 | 1.569201326 | high |
| GSM2328970 | 10.56193403 | 1.569201326 | high |
| GSM2329090 | 10.5720244 | 1.570700466 | high |
| GSM2329119 | 10.58205752 | 1.572191101 | high |
| GSM2329109 | 10.58575295 | 1.572740136 | high |
| GSM2328997 | 10.61875878 | 1.577643858 | high |
| GSM2328994 | 10.62221077 | 1.578156725 | high |
| GSM2329121 | 10.63493101 | 1.580046589 | high |
| GSM2328949 | 10.63857211 | 1.580587552 | high |
| GSM2329118 | 10.77182092 | 1.600384515 | high |
| GSM2328919 | 10.79519671 | 1.603857488 | high |
| GSM2329102 | 10.87764314 | 1.61610667 | high |
| GSM2329083 | 10.91437609 | 1.621564136 | high |
| GSM2328925 | 11.38915981 | 1.692103418 | high |
| GSM2329069 | 11.42148718 | 1.69690634 | high |

**Supplementary material Table S4. KEGG pathways analysis showed that the positive categories with the high levels of FCER1G**

| **NAME** | **SIZE** | **ES** | **NES** | **NOM p-val** | **FDR q-val** |
| --- | --- | --- | --- | --- | --- |
| KEGG_LYSOSOME | 101 | 0.758804 | 3.158945 | 0 | 0 |
| KEGG_CYTOKINE_CYTOKINE_RECEPTOR_INTERACTION | 211 | 0.600714 | 2.689862 | 0 | 0 |
| KEGG_GRAFT_VERSUS_HOST_DISEASE | 33 | 0.728484 | 2.401384 | 0 | 0 |
| KEGG_TYPE_I_DIABETES_MELLITUS | 37 | 0.701461 | 2.368781 | 0 | 0 |
| KEGG_ALLOGRAFT_REJECTION | 31 | 0.698586 | 2.321615 | 0 | 0 |
| KEGG_LEISHMANIA_INFECTION | 61 | 0.615444 | 2.321104 | 0 | 0 |
| **KEGG_ANTIGEN_PROCESSING_AND_PRESENTATION** | **67** | **0.593139** | **2.298418** | **0** | **0** |
| KEGG_CELL_ADHESION_MOLECULES_CAMS | 100 | 0.550617 | 2.290458 | 0 | 0 |
| KEGG_TOLL_LIKE_RECEPTOR_SIGNALING_PATHWAY | 88 | 0.579209 | 2.285831 | 0 | 0 |
| KEGG_COMPLEMENT_AND_COAGULATION_CASCADES | 61 | 0.610289 | 2.285272 | 0 | 0 |
| KEGG_NATURAL_KILLER_CELL_MEDIATED_CYTOTOXICITY | 111 | 0.548852 | 2.268546 | 0 | 0 |
| KEGG_NOD_LIKE_RECEPTOR_SIGNALING_PATHWAY | 49 | 0.609069 | 2.211645 | 0 | 0 |
| KEGG_GLUTATHIONE_METABOLISM | 35 | 0.637211 | 2.138262 | 0 | 2.67E-04 |
| KEGG_AUTOIMMUNE_THYROID_DISEASE | 41 | 0.609682 | 2.131376 | 0 | 2.48E-04 |
| KEGG_ECM_RECEPTOR_INTERACTION | 68 | 0.552297 | 2.114882 | 0 | 3.25E-04 |
| KEGG_CHEMOKINE_SIGNALING_PATHWAY | 148 | 0.481968 | 2.096068 | 0 | 3.99E-04 |
| KEGG_VIBRIO_CHOLERAE_INFECTION | 44 | 0.593762 | 2.092335 | 0 | 3.75E-04 |
| KEGG_PPAR_SIGNALING_PATHWAY | 52 | 0.568357 | 2.091233 | 0 | 3.55E-04 |
| KEGG_EPITHELIAL_CELL_SIGNALING_IN_HELICOBACTER_PYLORI_INFECTION | 58 | 0.560415 | 2.084784 | 0.002074689 | 4.16E-04 |
| KEGG_SYSTEMIC_LUPUS_ERYTHEMATOSUS | 45 | 0.584823 | 2.083017 | 0 | 3.95E-04 |
| KEGG_ARACHIDONIC_ACID_METABOLISM | 37 | 0.611716 | 2.072441 | 0 | 3.76E-04 |
| KEGG_HEMATOPOIETIC_CELL_LINEAGE | 79 | 0.520201 | 2.040629 | 0 | 7.67E-04 |
| KEGG_VIRAL_MYOCARDITIS | 58 | 0.517416 | 1.94033 | 0 | 0.0015658 |
| KEGG_GLYCOSAMINOGLYCAN_DEGRADATION | 16 | 0.700379 | 1.905806 | 0.002237137 | 0.0020568 |
| KEGG_AMINO_SUGAR_AND_NUCLEOTIDE_SUGAR_METABOLISM | 29 | 0.597406 | 1.880072 | 0 | 0.0028567 |
| KEGG_JAK_STAT_SIGNALING_PATHWAY | 127 | 0.44703 | 1.878882 | 0 | 0.0028038 |
| KEGG_INTESTINAL_IMMUNE_NETWORK_FOR_IGA_PRODUCTION | 39 | 0.54293 | 1.877646 | 0 | 0.0027 |
| KEGG_SNARE_INTERACTIONS_IN_VESICULAR_TRANSPORT | 29 | 0.579652 | 1.869313 | 0.002183406 | 0.0028606 |
| KEGG_PORPHYRIN_AND_CHLOROPHYLL_METABOLISM | 21 | 0.609134 | 1.809669 | 0 | 0.0056005 |
| KEGG_PROTEASOME | 38 | 0.519899 | 1.772095 | 0.008928572 | 0.007978 |
| KEGG_CYTOSOLIC_DNA_SENSING_PATHWAY | 39 | 0.500829 | 1.744803 | 0 | 0.010599 |
| KEGG_FOCAL_ADHESION | 166 | 0.385124 | 1.705884 | 0 | 0.0153013 |
| KEGG_ETHER_LIPID_METABOLISM | 17 | 0.610847 | 1.705018 | 0.004219409 | 0.0151261 |
| KEGG_SPHINGOLIPID_METABOLISM | 19 | 0.598727 | 1.687287 | 0.019067796 | 0.0171989 |
| KEGG_APOPTOSIS | 84 | 0.416172 | 1.667171 | 0.002123142 | 0.0197853 |
| KEGG_TRYPTOPHAN_METABOLISM | 33 | 0.484294 | 1.615997 | 0.010775862 | 0.0306649 |
| KEGG_ASTHMA | 24 | 0.523668 | 1.602324 | 0.018404908 | 0.0332834 |
| KEGG_NICOTINATE_AND_NICOTINAMIDE_METABOLISM | 17 | 0.572297 | 1.596152 | 0.013888889 | 0.03398 |
| KEGG_DRUG_METABOLISM_OTHER_ENZYMES | 28 | 0.49248 | 1.592749 | 0.017167382 | 0.0341556 |
| KEGG_REGULATION_OF_ACTIN_CYTOSKELETON | 165 | 0.362092 | 1.591742 | 0 | 0.0334951 |
| KEGG_GLYCEROLIPID_METABOLISM | 28 | 0.499433 | 1.590457 | 0.022540983 | 0.0329299 |
| KEGG_OXIDATIVE_PHOSPHORYLATION | 74 | 0.402584 | 1.576154 | 0 | 0.0360625 |
| KEGG_PRION_DISEASES | 31 | 0.487563 | 1.572034 | 0.015118791 | 0.0363896 |
| KEGG_T_CELL_RECEPTOR_SIGNALING_PATHWAY | 95 | 0.38725 | 1.559622 | 0.002083333 | 0.0400668 |
| KEGG_MAPK_SIGNALING_PATHWAY | 211 | 0.338527 | 1.541852 | 0 | 0.0447678 |
| KEGG_ENDOCYTOSIS | 125 | 0.365171 | 1.522206 | 0.004201681 | 0.0516822 |
| KEGG_PATHWAYS_IN_CANCER | 271 | 0.323137 | 1.512368 | 0 | 0.0549905 |
| KEGG_NEUROACTIVE_LIGAND_RECEPTOR_INTERACTION | 204 | 0.321114 | 1.461081 | 0.001964637 | 0.0800836 |
| KEGG_RIG_I_LIKE_RECEPTOR_SIGNALING_PATHWAY | 57 | 0.377115 | 1.415825 | 0.046747968 | 0.1090605 |
| KEGG_ADIPOCYTOKINE_SIGNALING_PATHWAY | 59 | 0.380591 | 1.413007 | 0.042462844 | 0.1091076 |
| KEGG_LEUKOCYTE_TRANSENDOTHELIAL_MIGRATION | 93 | 0.345146 | 1.403252 | 0.027310925 | 0.1139458 |
| KEGG_GALACTOSE_METABOLISM | 21 | 0.457414 | 1.391474 | 0.09307359 | 0.12173 |
| KEGG_BLADDER_CANCER | 39 | 0.407001 | 1.387819 | 0.06889353 | 0.1225311 |
| KEGG_MELANOMA | 57 | 0.368776 | 1.372536 | 0.057654075 | 0.1332535 |
| KEGG_METABOLISM_OF_XENOBIOTICS_BY_CYTOCHROME_P450 | 44 | 0.382503 | 1.344692 | 0.08350731 | 0.1568592 |
| KEGG_DRUG_METABOLISM_CYTOCHROME_P450 | 48 | 0.367978 | 1.340806 | 0.077922076 | 0.1573691 |
| KEGG_STARCH_AND_SUCROSE_METABOLISM | 26 | 0.424636 | 1.309459 | 0.12184874 | 0.1885503 |
| KEGG_ARRHYTHMOGENIC_RIGHT_VENTRICULAR_CARDIOMYOPATHY_ARVC | 64 | 0.345769 | 1.30518 | 0.072874494 | 0.1903646 |
| KEGG_CITRATE_CYCLE_TCA_CYCLE | 26 | 0.420028 | 1.299594 | 0.14078675 | 0.1931908 |
| KEGG_GLYCOLYSIS_GLUCONEOGENESIS | 53 | 0.352786 | 1.272455 | 0.10208333 | 0.2232435 |
| KEGG_FRUCTOSE_AND_MANNOSE_METABOLISM | 26 | 0.409661 | 1.271955 | 0.1626506 | 0.220208 |
| KEGG_PEROXISOME | 59 | 0.339959 | 1.265776 | 0.12258065 | 0.2247189 |
| KEGG_MELANOGENESIS | 74 | 0.324973 | 1.264404 | 0.08631579 | 0.2227436 |
| KEGG_HISTIDINE_METABOLISM | 22 | 0.427132 | 1.254674 | 0.17910448 | 0.2322457 |
| KEGG_PATHOGENIC_ESCHERICHIA_COLI_INFECTION | 45 | 0.351468 | 1.243494 | 0.14730291 | 0.2442303 |
| KEGG_VEGF_SIGNALING_PATHWAY | 60 | 0.325372 | 1.230672 | 0.14344262 | 0.2590717 |
| KEGG_ARGININE_AND_PROLINE_METABOLISM | 41 | 0.356434 | 1.216429 | 0.17834395 | 0.2762626 |
| KEGG_ALZHEIMERS_DISEASE | 116 | 0.286341 | 1.202756 | 0.122 | 0.2931489 |
| KEGG_BETA_ALANINE_METABOLISM | 19 | 0.401737 | 1.177643 | 0.25 | 0.330132 |
| KEGG_GAP_JUNCTION | 71 | 0.303481 | 1.175479 | 0.1923077 | 0.3291427 |
| KEGG_DILATED_CARDIOMYOPATHY | 74 | 0.300118 | 1.174483 | 0.18181819 | 0.3261372 |
| KEGG_AXON_GUIDANCE | 105 | 0.284769 | 1.165753 | 0.15737052 | 0.3359929 |
| KEGG_FC_GAMMA_R_MEDIATED_PHAGOCYTOSIS | 77 | 0.296412 | 1.161024 | 0.197556 | 0.3393628 |
| KEGG_ABC_TRANSPORTERS | 33 | 0.346885 | 1.155954 | 0.23966943 | 0.3432618 |
| KEGG_ACUTE_MYELOID_LEUKEMIA | 51 | 0.312821 | 1.147881 | 0.24696356 | 0.3522186 |
| KEGG_GLYCOSPHINGOLIPID_BIOSYNTHESIS_LACTO_AND_NEOLACTO_SERIES | 20 | 0.379146 | 1.13921 | 0.26875 | 0.3628479 |
| KEGG_THYROID_CANCER | 25 | 0.366215 | 1.138727 | 0.2857143 | 0.3591927 |
| KEGG_BASAL_CELL_CARCINOMA | 36 | 0.338397 | 1.127103 | 0.27489176 | 0.3752137 |
| KEGG_TIGHT_JUNCTION | 96 | 0.27779 | 1.124218 | 0.23408625 | 0.375672 |
| KEGG_STEROID_HORMONE_BIOSYNTHESIS | 33 | 0.340527 | 1.113022 | 0.28450108 | 0.391506 |
| KEGG_GLYCOSAMINOGLYCAN_BIOSYNTHESIS_HEPARAN_SULFATE | 17 | 0.394905 | 1.108271 | 0.32765958 | 0.3958142 |
| KEGG_PANCREATIC_CANCER | 66 | 0.291451 | 1.10023 | 0.2993763 | 0.405749 |
| KEGG_CALCIUM_SIGNALING_PATHWAY | 145 | 0.248118 | 1.074223 | 0.28118393 | 0.4510498 |
| KEGG_GLYCEROPHOSPHOLIPID_METABOLISM | 43 | 0.305843 | 1.072435 | 0.34599155 | 0.4493177 |
| KEGG_HYPERTROPHIC_CARDIOMYOPATHY_HCM | 69 | 0.274971 | 1.068915 | 0.30588236 | 0.4507963 |
| KEGG_PYRUVATE_METABOLISM | 33 | 0.320752 | 1.067673 | 0.34763947 | 0.4483309 |
| KEGG_AMYOTROPHIC_LATERAL_SCLEROSIS_ALS | 47 | 0.299977 | 1.065439 | 0.3263158 | 0.4480473 |
| KEGG_FC_EPSILON_RI_SIGNALING_PATHWAY | 63 | 0.278515 | 1.043344 | 0.38543898 | 0.4874022 |
| KEGG_VASCULAR_SMOOTH_MUSCLE_CONTRACTION | 81 | 0.253253 | 1.004827 | 0.44421905 | 0.565607 |
| KEGG_GNRH_SIGNALING_PATHWAY | 74 | 0.25065 | 0.99057 | 0.48015872 | 0.5920585 |
| KEGG_FATTY_ACID_METABOLISM | 36 | 0.294785 | 0.983188 | 0.46843177 | 0.6025962 |
| KEGG_NITROGEN_METABOLISM | 21 | 0.329217 | 0.973318 | 0.5010707 | 0.6184616 |
| KEGG_REGULATION_OF_AUTOPHAGY | 27 | 0.306601 | 0.966088 | 0.49779737 | 0.6287636 |
| KEGG_ALDOSTERONE_REGULATED_SODIUM_REABSORPTION | 36 | 0.281517 | 0.949069 | 0.5386266 | 0.6615321 |
| KEGG_TGF_BETA_SIGNALING_PATHWAY | 77 | 0.2327 | 0.910862 | 0.62425447 | 0.7430484 |
| KEGG_GLYCINE_SERINE_AND_THREONINE_METABOLISM | 23 | 0.290545 | 0.887624 | 0.6385809 | 0.7907153 |
| KEGG_RENAL_CELL_CARCINOMA | 58 | 0.237696 | 0.887181 | 0.6757322 | 0.7836434 |
| KEGG_ADHERENS_JUNCTION | 61 | 0.237602 | 0.884697 | 0.6942675 | 0.7811449 |
| KEGG_LYSINE_DEGRADATION | 26 | 0.281435 | 0.873107 | 0.64615387 | 0.7997332 |
| KEGG_PROXIMAL_TUBULE_BICARBONATE_RECLAMATION | 19 | 0.297097 | 0.86974 | 0.6515151 | 0.7990363 |
| KEGG_CHRONIC_MYELOID_LEUKEMIA | 66 | 0.228693 | 0.86301 | 0.7298387 | 0.8050798 |
| KEGG_TYPE_II_DIABETES_MELLITUS | 43 | 0.238333 | 0.835623 | 0.76059324 | 0.8530002 |
| KEGG_VASOPRESSIN_REGULATED_WATER_REABSORPTION | 37 | 0.228331 | 0.775956 | 0.8126273 | 0.9495658 |
| KEGG_TYROSINE_METABOLISM | 34 | 0.234151 | 0.771631 | 0.84243697 | 0.9473106 |
| KEGG_CARDIAC_MUSCLE_CONTRACTION | 55 | 0.203485 | 0.747745 | 0.89555126 | 0.969995 |
| KEGG_LINOLEIC_ACID_METABOLISM | 15 | 0.279123 | 0.736701 | 0.8333333 | 0.9743984 |
| KEGG_LONG_TERM_DEPRESSION | 51 | 0.200655 | 0.733914 | 0.9089069 | 0.9684677 |
| KEGG_ERBB_SIGNALING_PATHWAY | 75 | 0.183206 | 0.722926 | 0.9597586 | 0.9709634 |
| KEGG_PROGESTERONE_MEDIATED_OOCYTE_MATURATION | 64 | 0.185501 | 0.700029 | 0.95670104 | 0.9818317 |
| KEGG_SELENOAMINO_ACID_METABOLISM | 15 | 0.244646 | 0.674158 | 0.9224319 | 0.9912038 |
| KEGG_RETINOL_METABOLISM | 31 | 0.196634 | 0.640048 | 0.9710744 | 0.9992594 |
| KEGG_DORSO_VENTRAL_AXIS_FORMATION | 16 | 0.206016 | 0.569639 | 0.96629214 | 1 |
| KEGG_BUTANOATE_METABOLISM | 26 | 0.185229 | 0.567016 | 0.9867257 | 0.9998218 |
| KEGG_OLFACTORY_TRANSDUCTION | 59 | 0.091246 | 0.351357 | 1 | 1 |
